# Neuronal allocation and sparse coding of episodic memories in the human hippocampus

**DOI:** 10.1038/s41598-025-21967-7

**Published:** 2025-11-18

**Authors:** Catherine W. Tallman, Peter N. Steinmetz, John T. Wixted

**Affiliations:** 1 Department of Psychology, University of California San Diego, San Diego, CA USA; 2 Neurtex Brain Research Institute, Dallas, TX USA

**Keywords:** Sparse coding, Episodic memory, Human hippocampus, Single-unit recording, Neuronal allocation, Subsequent memory, Cognitive neuroscience, Human behaviour

## Abstract

Neurocomputational models hold that individual episodic memories are represented by a sparse, pattern-separated coding scheme in the hippocampus. Animal studies further suggest that the allocation of neurons to such codes is non-random and may be biased by their excitability at the time of encoding. Here, utilizing an independent dataset of single-unit recordings from epilepsy patients, we report that only remembered items that elicited a relative increase in firing at encoding were associated with a sparse, pattern-separated neural code at retrieval, and this effect was specific to the hippocampus. These findings provide evidence in humans that remembered episodic memories are represented by sparse codes in the hippocampus. Although excitability is not typically defined in terms of firing rates, the present results raise the possibility that excitability at encoding may influence the likelihood of recruitment into such codes.

**Supplementary Information:**

The online version contains supplementary material available at 10.1038/s41598-025-21967-7.

## Introduction

The structures of the medial temporal lobe (MTL), particularly the hippocampus, support the encoding, consolidation, and retrieval of declarative memory^1–5^. Damage to the MTL, critically the hippocampus, impairs the formation of new memories and the retrieval of recently encoded memories^6–9^. This suggests that the hippocampus is necessary for declarative memory, unlike lesions to other regions such as the amygdala^10^. One form of declarative memory, episodic memory, refers to the ability to recollect a single event within its original spatial and temporal context^4,11,12^. Episodic memories, by definition, are formed rapidly, and they are often studied using experimental paradigms that involve single-trial learning. Here, we focus on how individual episodic memories are represented by sparse codes in the human hippocampus, and how their subsequent retrieval is related to the spiking activity of single neurons during encoding.

Neurocomputational models hold that episodic memories are represented in the hippocampus according to sparse, distributed, and pattern-separated codes^13–16^. In a sparse coding scheme, a single episodic memory is associated with relatively few hippocampal neurons (lifetime sparseness), and each neuron is associated with relatively few individual episodic memories (population sparseness). Moreover, individual episodic memories are theoretically encoded in sparse distributed assemblies to reduce catastrophic interference^14,17,18^, protecting against massive forgetting that would otherwise occur if episodic memories were fully distributed across the population of hippocampal neurons. The existence of sparse coding is supported by early-gene studies in animals^19–22^ and by single-unit^23–25^ and fMRI work in humans^26,27^. However, other theories hold that humans encode episodic memories differently than animals, such that memories are represented by partially overlapping neural assemblies built with shared concept representations^28^. Single-neuron recordings from the human hippocampus during an episodic memory task offer a unique methodological approach to test these competing perspectives.

A critical mechanistic question concerns how specific neurons are assigned to these sparse hippocampal codes at the time of encoding. The neuronal allocation hypothesis proposes that recruitment is non-random and reflects differences in a neuron’s likelihood of activation during learning^29–32^. This likelihood is influenced by intrinsic factors such as cAMP Response Element–Binding (CREB) protein dependent increases in excitability^19–22,33,34^. These increases bias neuronal allocation, as demonstrated by direct CREB manipulations^35^. In addition, allocation can be influenced by learning-induced changes in excitability^36^, or by synaptic factors such as connectivity, prior plasticity, and momentary synaptic drive^37,38^. Studies in rodents demonstrate this biased recruitment in both the amygdala during fear conditioning and the hippocampus during contextual learning^19,20,36^. In humans, direct measures of excitability are not feasible, and the firing rate of a neuron during encoding might serve as an observable correlate of the likelihood of activation. It is, however, an indirect index that cannot definitively disentangle contributions of pre-existing levels of intrinsic excitability, learning-induced excitability, and synaptic factors.

Findings of sparse coding and neuronal allocation in animals raise the question of whether similar principles govern episodic memory coding in the human hippocampus, a question that we address through single-unit recordings in the human MTL. In the laboratory, episodic memory is often operationalized in terms of recognizing items from a recently presented list, where the participant’s task is to distinguish between previously encountered old items from the list (targets) and newly presented items (foils). The neural episodic memory signal should reflect activity that is preferentially associated with old items compared to new items, but should also be (1) specific to one or, perhaps, a few old items from the list (i.e., lifetime sparseness), (2) specific to a small assembly of neurons (i.e., population sparseness), (3) preferentially associated with relatively active neurons during encoding (either already spiking at item onset or showing a relative increase in firing), consistent with firing as an indirect correlate of excitability-based allocation, and (4) specific to the hippocampus. Taken together, these predictions offer an unusually precise test of theoretical and computational models.

However, many human studies report neural signals that diverge from these predictions. The predicted sparse, item-specific, episodic memory signal contrasts with the neural signature of the more commonly investigated *generic* memory signal, whereby neurons respond differentially to the entire category of old items vs. new items. This generic episodic memory signal is not item-specific, and it has been observed not only in the hippocampus but also in the amygdala, anterior cingulate, prefrontal cortex, and parietal cortex^23,39–41^. Given that this signal is both generic and ubiquitous, it may not be the fundamental episodic memory signal that neurocomputational models have long predicted.

Prior studies have reported evidence of sparsely coded episodic memories in the human hippocampus using single-unit recordings but were limited to one task and to one research group^23–25^. Here, we used an independent, publicly available dataset from another research group to test the predictions of neurocomputational models of episodic memory representation^42,43^. This dataset differed from the previous dataset in ways that should not influence the predictions of neurocomputational models. Specifically, the dataset differed in the episodic memory task design (old/new recognition vs. continuous recognition), stimulus material (images vs. words), and spike sorting algorithm. Thus, it was an opportunity to replicate prior work in an independent dataset with several experimental differences that would still exhibit the sparsely coded item-specific episodic memory signal. More importantly, this dataset also afforded the opportunity to investigate the relationships among sparse coding, neuronal allocation, and subsequent memory.

## Results

### Behavioral performance

Participants performed above chance on the recognition memory test for old items (targets), 67.7% correct, SEM = 0.2% (i.e., hit rate = 0.677) and for new items (foils), 73.9% correct, SEM = 0.2% (i.e., false alarm rate = 1–0.739 = 0.261). For each session *d’* was calculated as *z*(false alarm rate) – *z*(hit rate). The average *dʹ* across sessions was 1.24 (SEM = 0.002, min = 0.05, max = 2.47). Sessions with no hippocampal or amygdala units (*N* = 4) and sessions with negative *d’* values (*N* = 4) were excluded, resulting in 79 sessions from 55 participants (µ_age_ = 37.2 years; Female: *N* = 23, Male: *N* = 32) included in this report.

### Item-specific episodic memory signal selectively detected in the hippocampus at retrieval

A sparse episodic memory signal was defined as a small fraction of neurons selectively responding to a small fraction of “old” items, the items studied before the retrieval phase of the recognition memory test. In the simplest case, a single neuron would exhibit a strong response to a single old item but not to any other old item, nor to any new item. In other words, the neuron’s response would be (1) item-specific, and (2) specific to that item only if it is old. Conversely, the generic signal is (by definition) not item-specific and instead consists of a difference in the average population or single neuron firing rate in response to either old or new items. We investigated the presence of the item-specific sparse coding signal by comparing the full distributions of item-by-neuron spike counts for targets vs. foils at retrieval.

**Fig. 1:**
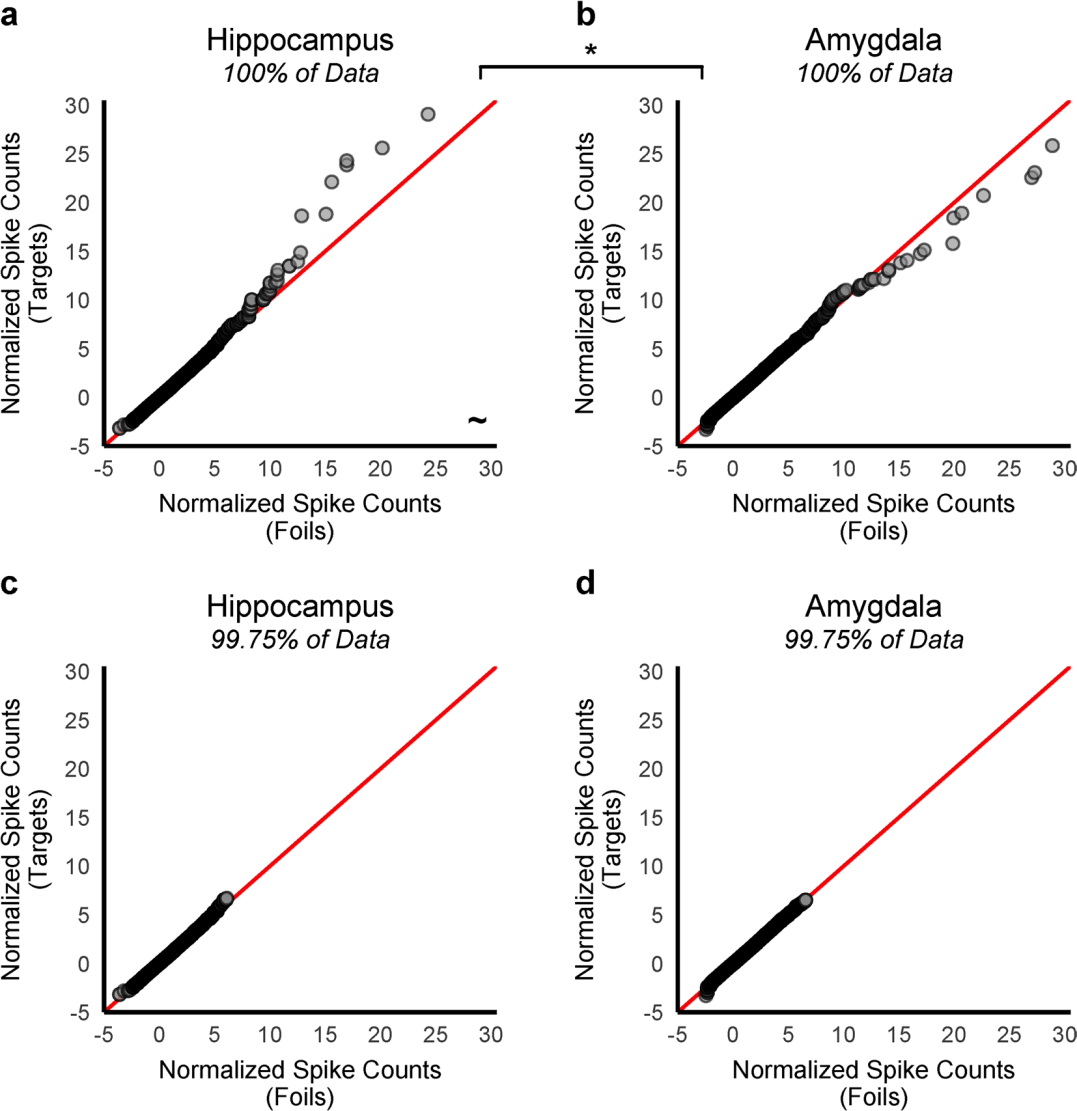
Empirical quantile-quantile (Q-Q) plots of the target- vs. foil-by-neuron normalized spike-count distributions at retrieval. The target-by-neuron response distribution (y-axis) vs. the foil-by-neuron response distribution (x-axis) was compared separately for the hippocampus (**a**, **c**) and amygdala (**b**, **d**). The top panels (**a**, **b**) plot the distributions of 100% of the normalized spike counts from each neuron in response to each retrieval trial (item presentation), across all patients (*N* = 79) and sessions (*N* = 55), in the hippocampus (**a**: 72,538 recordings from 736 neurons) and amygdala (**b**: 99,944 recordings from 1,038 neurons). Densely grouped points (dark gray) represent thousands of recordings, and less densely grouped points (light gray) represent relatively few recordings. In panel (**a**), the nonlinear upward deflection toward the y-axis reflected that the hippocampal target-by-neuron response distribution had a different shape than the foil-by-neuron response distribution, and specifically, a more positive skew. The deflection in (**a**) is predicted by a sparse coding account, which further predicts that the deflection reflects the strong responses of a small percentage of neurons in response to target items. A less pronounced deflection in the opposite direction was visible in the amygdala (**b**). After removing the top 0.25% of the target and foil spike counts for the hippocampus (**c**) and the amygdala (**d**), the deflections in (**a**) and (**b**) were no longer apparent. The skewness values of the two normalized spike count distributions (targets vs. foils) were compared to determine if the theoretically predicted deflection in panel (**a**) was statistically reliable using bootstrap tests (B = 10,000, *p* < 0.05, one-tailed). The difference in skewness between the target- vs. foil-response distributions was significantly different between the brain regions (hippocampus vs. amygdala). In the hippocampus only, skewness was greater for targets than foils, but this difference did not reach significance (*p* = 0.070). Refer to Table 1 for detailed statistical reporting. *p < 0.05, ~*p* < 0.10, one-tailed.

We used empirical quantile-quantile (Q-Q) plots to visualize the shapes of the normalized spike count distributions at retrieval, consisting of each neuron’s response to each item, across all sessions and participants. In this type of plot, the quantiles of one dataset are plotted on one axis, and the corresponding quantiles of the second dataset are plotted on the other axis. We assessed the presence of the sparse coding signal by comparing the quantiles of the foil-by-neuron normalized spike count distribution (x-axis) vs. the quantiles of the target-by-neuron normalized spike count distribution (y-axis), separately for the hippocampus and amygdala (Fig. 1a, b). Deviations from linearity arise when the distributions differ in moments beyond the first (mean) and second (standard deviation). Of most interest here, an upward deflection away from the diagonal at the upper right of the Q-Q plot indicates a difference in the third moment (skewness). More specifically, it indicates that the target item distribution is more positively skewed than the foil item distribution (a feature that might not be visually apparent in a plot of the frequency distributions). Of note, points on a Q-Q plot that are densely grouped represent thousands of recordings that have similar values for both distributions, so there are many more points on the plot than it appears. A detailed schematic and explanation of how to interpret these Q-Q plots is provided in the Methods and Supplementary Fig. S8.

In the hippocampus, an upward deflection was evident at higher quantiles, indicating the target (i.e., old item) distribution was more positively skewed compared to the foil (i.e., new item) distribution (Fig. 1a). Removing the top 0.25% of the highest firing item-by-neuron recordings from both distributions eliminated this deflection (Fig. 1c). This demonstrated there was higher spiking for a small proportion of hippocampal neurons in response to few items in the target distribution, supporting a sparse coding interpretation. In the amygdala, a less pronounced deflection was observed in the opposite direction (Fig. 1b). Similarly, after we removed the top 0.25% of the highest firing recordings from both distributions, the deflection toward the x-axis was no longer apparent (Fig. 1d).

We then tested whether the visual deflections were statistically significant by focusing on the skewness measures of the two distributions (Table 1). First, and most importantly, we tested for an interaction between the two regions of interest (hippocampus and amygdala) and the skewness of the two distributions (target and foils) using bootstrap tests. Theoretically, the sparse coding signal would be evident as an interaction effect such that the magnitude of the target–minus-foil difference in skewness would be larger in the hippocampus than in the amygdala. A bootstrap test (B = 10,000, *p* < 0.05, one-tailed) confirmed a significant interaction between MTL region (hippocampus vs. amygdala) and distribution (target vs. foil) in skewness (*p* = 0.049), replicating a key finding reported by Urgolites et al. (2022).

**Table 1:** Comparison of statistical moments between target vs. foil distributions at retrieval.

	Interaction	Region					
	∆ (H − A) Target–Foil	Hippocampus			Amygdala		
	*p*	Targets	Foils	*p*	Targets	Foils	*p*
Mean	0.047*	0.08	0.08	0.334	0.14	0.16	0.019*
SD	0.059	1.22	1.18	0.054	1.22	1.23	0.344
Skewness	0.049*	4.17	3.05	0.070	3.00	3.28	0.254
Kurtosis	0.052	58.0	29.9	0.086	26.0	34.9	0.177

A significant interaction was defined as a greater difference in the value of a given statistical moment (e.g., skewness) between the target- vs. foil- normalized spike count distributions in the hippocampus (72,538 recordings from 736 neurons) compared to the corresponding difference in the amygdala (99,944 recordings from 1,038 neurons), according to bootstrap tests (Δ(H–A) Target–Foil, B = 10,000, *p* < 0.05, one-tailed). The interactions for mean (*p* = 0.047) and skewness (*p* = 0.049) were significant. Differences in each statistical moment between the two distributions (target vs. foil) were also considered separately within each structure (hippocampus and amygdala) using bootstrap tests (B = 10,000, *p* < 0.05, one-tailed). In the hippocampus, differences in the upper moments of the target and foil spike count distributions were greater for targets, but did not reach significance (standard deviation: *p* = 0.054, skewness: *p* = 0.070, kurtosis: *p* = 0.086). In the amygdala, the differences were greater for foils compared to targets, but also did not reach significance (standard deviation: *p* = 0.344, skewness: *p* = 0.254, kurtosis: *p* = 0.177). Mean firing in the amygdala distinguished between target and foil items (*p* = 0.019) but not in the hippocampus (*p* = 0.334). *p < 0.05, one-tailed.

Additional bootstrap tests were conducted to determine the directionality of the effect (B = 10,000, *p* < 0.05, one-tailed). In the hippocampus, skewness was greater for the target-by-neuron distribution (4.17) than for the foil-by-neuron distribution (3.05), though the difference did not reach significance (*p* = 0.070). In the amygdala, skewness was slightly lower for targets (3.00) than foils (3.28), but this difference was not significant (*p* = 0.254). A significant difference in mean firing between targets and foils was only present in the amygdala (0.14 vs. 0.16, *p* = 0.019), consistent with the generic memory signal identified in previous work^23,41^ (see Supplementary Material).

### Item-specific episodic memory signal selectively detected in the hippocampus at retrieval for targets that elicited a relative increase in firing at encoding

We next tested whether a relative increase in firing at encoding biased neurons to be assigned to a sparse code, a prediction informed by allocation models in which excitability influences neuronal recruitment. Each target was classified by a median split on firing rates pre- vs. post-stimulus presentation at encoding (Low–High, High–High, High–Low, Low–Low). Guided by predictions from sparse coding and inspired by allocation theory, we expected the item-specific episodic memory signal at retrieval would be selective to the hippocampus and to only occur for targets that were relatively active at encoding, either through high baseline firing (High–High) or through relative increased firing at stimulus onset (Low–High). Additionally, we expected that the episodic memory signal would remain undetected in the amygdala at retrieval (Fig. 1; Table 1), regardless of spiking observed before and during item presentation at encoding.

**Fig. 2:**
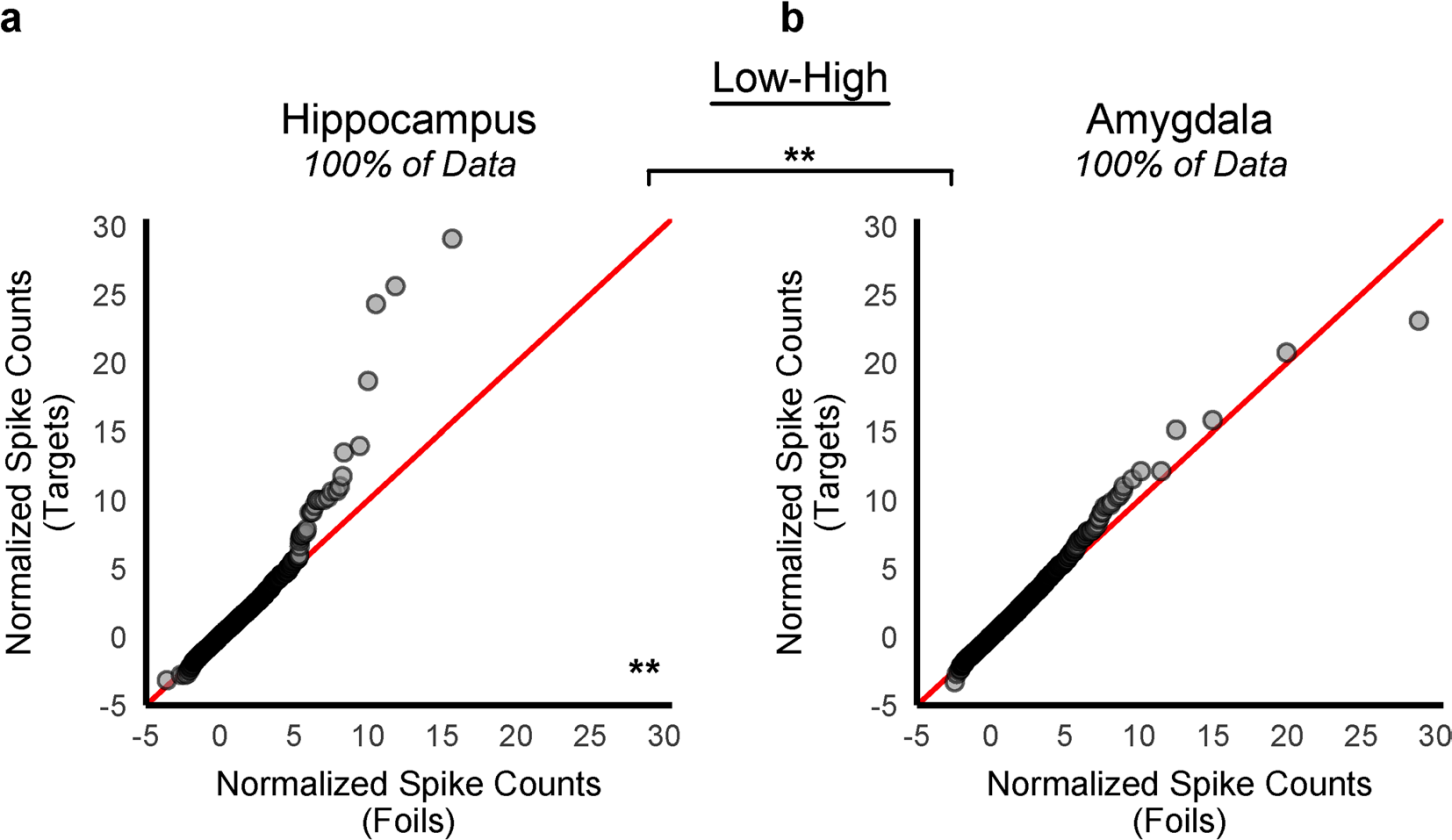
Empirical quantile-quantile (Q–Q) plots of the normalized spike count distributions at retrieval partitioned by relative excitability at encoding. The target-by-neuron response distribution (y-axis) vs. the foil-by-neuron response distributions (x-axis) was compared for items categorized by their pattern of spiking activity at encoding for the hippocampus (**a**) and the amygdala (**b**). As in Fig. 1, the distributions consisted of normalized spike counts at retrieval from each neuron in response to each trial across all patients and sessions. Targets were divided into distinct subsets based on their relative excitability during encoding (spiking activity directly before and during stimulus presentation): Low–High,  High–High, High–Low, and Low–Low. For each category, the foil-by-neuron response distribution (x-axis) was compared to the corresponding target-by-neuron response distribution in the hippocampus and amygdala. The predicted sparse coding signal (nonlinear deflection toward the y-axis) was selectively detected in the hippocampus for items at retrieval with a relative increase in excitability at encoding (**a**: Low-High, 43,577 recordings) and was not present for the amygdala (b: Low-High, 59,661 recordings). The deflection was not present for any other pattern of firing at encoding in the hippocampus and amygdala (see Supplementary Fig. S1). The skewness values between the two distributions (Low-High targets vs. foils) differed significantly between brain regions (hippocampus vs. amygdala), with a significant difference observed in the hippocampus only (B = 10,000, *p* < 0.0125, Bonferroni-corrected, two-tailed). Refer to Table 2 for detailed statistical reporting. Supplementary Fig. S2 reports the corresponding Q-Q plots with the top 0.25% of both the target-by-neuron and foil-by-neuron distributions removed. The deflection observed in panel (**a**) disappeared after removing the top 0.25% of data, indicating that relatively few hippocampal neurons fired strongly in response to targets with a relative increase in excitability at encoding compared to foil items. **p < 0.01.

We repeated our statistical approach to identify the item-specific memory signal at retrieval for each encoding-defined category, beginning with a visual inspection of the relevant foil- and target- response distributions on Q-Q plots. A sharp upward deflection on the Q-Q plot (indicative of greater skewness for the target distribution relative to the foil distribution) was present only for the targets that were categorized as Low-High at encoding (Fig. 2) and was further selective to hippocampal neurons (Fig. 2a). In the amygdala, by contrast, almost all points were densely grouped along the red diagonal line (Fig. 2b), providing no evidence of a skewness difference between targets and foils at retrieval with a relative increase in excitability at encoding. The upward deflection was not visually apparent on any of the remaining Q-Q plots of the spike count distributions at retrieval, either in the hippocampus or the amygdala, for the subsets of targets in which spiking at encoding remained high (High-High), spiking decreased (High-Low), or remained low (Low-Low; see Supplementary Fig. S1 and Supplementary Fig. S2 for corresponding Q-Q plots). Bootstrap tests (B = 10,000, *p* < 0.0125, Bonferroni corrected, two-tailed) confirmed a significant interaction between the skewness values of the target (Low-High) and foil normalized spike count distributions at retrieval between the hippocampus and amygdala (Table 2, *p* = 0.008). In the hippocampus, skewness was significantly higher for Low-High targets than foils (6.79 vs. 3.05, *p* = 0.004) and this difference was not significant in the amygdala (3.27 vs. 3.28, *p* = 0.989). No other excitability patterns at encoding showed a reliable interaction for the skewness values or the other distribution moments (mean, SD, kurtosis) for Low-High targets (Supplementary Table S1).

**Table 2:** Statistical results for skewness of the target vs. foil distributions at retrieval by the pattern of firing at encoding.

	Skewness						
	Interaction	Region					
	Δ (H–A) Target–Foil	Hippocampus			Amygdala		
	*p*	Targets	Foils	*p*	Targets	Foils	*p*
Low–High	0.008**	6.79	3.05	0.004**	3.27	3.28	0.989
High–High	0.803	2.61	3.05	0.716	3.18	3.28	0.878
High–Low	0.891	2.21	3.05	0.527	2.64	3.28	0.388
Low–Low	0.473	3.10	3.05	0.965	2.42	3.28	0.173

A significant interaction was defined as a greater difference in a given statistical moment between one subset of targets partitioned by the pattern of firing at encoding (spiking activity directly before and during stimulus onset) vs. the foil normalized spike count distributions in one region (e.g., the hippocampus), compared to the corresponding difference in the other region (e.g., the amygdala), according to bootstrap tests (Δ(H–A) Target–Foil, B = 10,000, Bonferroni corrected, *p* < 0.0125, two-tailed). The interaction was significant for the subset of targets with a relative increase in excitability at encoding (Low-High: *p* = 0.008) but not for any other categories of targets (High-High: *p* = 0.803, High-Low: *p* = 0.891, or Low-Low: *p* = 0.473). Within the hippocampus, a significant difference between the skewness values of the target vs. foil distributions at retrieval (theoretically indicative of the item-specific memory signal) was observed only for targets with a relative increase in excitability at encoding (Low-High: *p* = 0.004). Within the amygdala, no significant difference in skewness between the target and foil distributions was observed. Corresponding analyses for the other statistical moments are reported in Supplementary Table 1. **p < 0.01.

### Item-specific episodic memory signal selectively detected in the hippocampus at retrieval for remembered but not forgotten targets

**Fig. 3:**
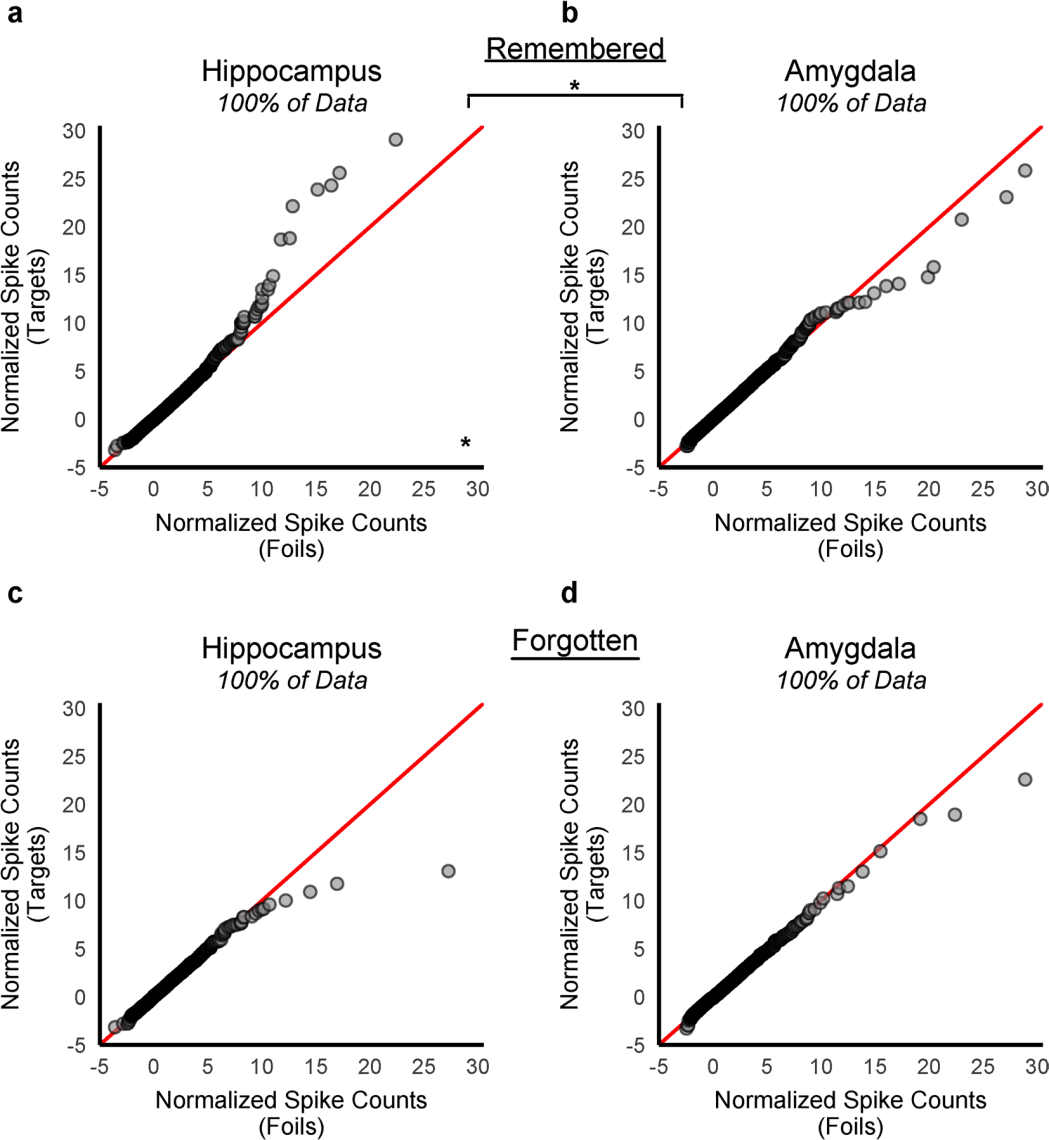
Empirical quantile–quantile (Q–Q) plots of the normalized spike count distributions at retrieval for remembered and forgotten items. As in Figs. 1 and 2, the distributions consisted of normalized spike counts from each neuron in response to each trial across all patients and sessions. Targets were divided based on their memory judgment: remembered (**a**, **b**) and forgotten (**c**, **d**). The foil-by-neuron response distribution (x-axis) was compared to corresponding target-by-neuron response distributions in the hippocampus (**a**: remembered, 60,991; **c**: forgotten, 47,787 recordings) and amygdala (**b**: remembered, 84,033; **d**: forgotten, 65,763 recordings). In panel (**a**), the sharp deflection toward the y-axis was evident for remembered targets in the hippocampus and a small deflection in the opposite direction was observed in the amygdala (**b**). This deflection was not present for forgotten items in the hippocampus (**c**) or the amygdala (**d**). The skewness values between the two distributions (remembered targets vs. foils) differed significantly between brain regions (hippocampus vs. amygdala), with a significant difference observed in the hippocampus only (B = 10,000, *p* < 0.05, two-tailed). No visual (Q–Q plots) or statistical evidence of a difference in skewness between the forgotten target vs. foil distributions was observed in either the hippocampus or amygdala. Refer to Table 3 for detailed statistical reporting. Supplementary Fig. S3 reports Q-Q plots for remembered and forgotten items with the top 0.25% of both the target-by-neuron and foil-by-neuron distributions removed. The deflection observed in panel (**a**) disappeared after removing the top 0.25% of data, indicating that relatively few neurons fired strongly in response to remembered targets compared to foil items, and only within the hippocampus. *p < 0.05.

Next, we generated Q-Q plots to compare the distributions of the normalized spike counts of remembered targets to foil items and forgotten targets to foil items, separately (Fig. 3). Within the hippocampus, greater skewness of the target-by-neuron distribution was apparent in the Q-Q plots when the targets were subsequently remembered (Fig. 3a) but not when they were forgotten (Fig. 3c). No apparent nonlinear trends were evident in the Q-Q plots for the amygdala whether the targets were remembered or forgotten (Fig. 3b, d). The deflection in the hippocampal Q-Q plot (Fig. 3a) disappeared after removing 0.25% of both the remembered target- and foil-by-neuron recordings (Supplementary Fig. S3), indicating relatively few high firing neurons contributed to the greater skewness of the remembered target-by-neuron distribution when compared to foil items.

Statistically, bootstrap tests (B = 10,000, *p* < 0.05, two-tailed) identified a significant interaction (*p* = 0.027) between the skewness of the two distributions (remembered targets vs. foils) and brain region (hippocampus vs. amygdala). Considering the hippocampus only, skewness was significantly greater for the remembered target distribution compared to the foil item distribution (4.82 vs. 3.05, *p* = 0.036). The interaction test for a difference in kurtosis of the normalized spike count distributions (remembered targets vs. foils) between the MTL regions (hippocampus vs. amygdala) was significant (*p* = 0.037) but was not significant for mean (*p* = 0.854) or standard deviation (*p* = 0.083). Within each region considered separately, a significant difference in the remembered vs. foil item distributions was also selectively observed in the hippocampus for standard deviation (1.26 vs. 1.18, *p* = 0.015). Similar effects in the hippocampus were not observed for forgotten targets, and no such effects were observed in the amygdala for either remembered or forgotten targets (Table 3).

**Table 3:** Comparison of statistical moments between remembered and forgotten target vs. foil distributions at retrieval.

Statistical moment	Subsequent memory	Interaction	Hippocampus			Amygdala		
		Δ (H–A) Target–Foil	Target	Foil	p	Target	Foil	p
Mean	Remembered	0.854	0.09	0.08	0.316	0.16	0.16	0.395
	Forgotten	<0.001***	0.07	0.08	0.470	0.09	0.16	< 0.001***
SD	Remembered	0.083	1.26	1.18	0.015*	1.24	1.23	0.634
	Forgotten	0.617	1.15	1.18	0.542	1.18	1.23	0.085
Skewness	Remembered	0.027*	4.82	3.05	0.036*	2.94	3.28	0.456
	Forgotten	0.603	2.30	3.05	0.478	3.15	3.28	0.834
Kurtosis	Remembered	0.037*	72.3	29.9	0.064	24.9	34.9	0.345
	Forgotten	0.720	12.8	29.9	0.545	28.8	34.9	0.660

A significant interaction was defined as a greater difference in a given statistical moment between the remembered or forgotten target- vs. foil- normalized spike count distributions in one region (e.g., the hippocampus), compared to the corresponding difference in the other region (e.g., the amygdala), according to bootstrap tests (Δ(H–A) Target–Foil, B = 10,000, *p* < 0.05) The interaction between the two distributions (remembered targets vs. foils) and brain regions (hippocampus vs. amygdala) was significant for skewness (*p* = 0.027) and kurtosis (*p* = 0.037). Within each region considered separately, significantly greater standard deviation (*p* = 0.015) and skewness (*p* = 0.036) values were observed for remembered targets compared to foils in the hippocampus. These effects were not present in the amygdala for remembered items or forgotten items in either the hippocampus or amygdala. *p < 0.05.

We additionally compared spike count distributions for remembered versus forgotten targets directly. Unlike prior analyses described thus far, foil item recordings were not included. The remembered target-by-neuron response distribution was plotted on the y-axis of the Q-Q plot while the forgotten target-by-neuron response distribution was plotted on the x-axis (Supplementary Fig. S4). The difference in skewness was selective to the hippocampus, both visually evident on the Q-Q plots and statistically confirmed via bootstrap tests (Supplementary Table S2). This direct comparison of remembered vs. forgotten targets provided further evidence linking sparse coding to subsequent memory. In sum, the sparse coding signal was detected in the hippocampus only for remembered items, relative to both foils and forgotten items.

### Item-specific episodic memory signal selectively detected in the hippocampus at retrieval for remembered targets that elicited a relative increase in firing at encoding

Thus far, we identified two theoretically motivated and distinct subsets of targets for which the target-by-neuron response distribution exhibited greater skewness than the foil-by-neuron response distribution: (1) targets with relatively increased firing at encoding and (2) targets that were subsequently remembered. Therefore, we posited that the item-specific episodic memory signal in the hippocampus (i.e., elevated skewness resulting from a small percentage of recordings) would be selectively identified for targets that *both* demonstrated low firing before stimulus onset and high firing during stimulus presentation (Low-High) at encoding and were subsequently remembered.

**Fig. 4:**
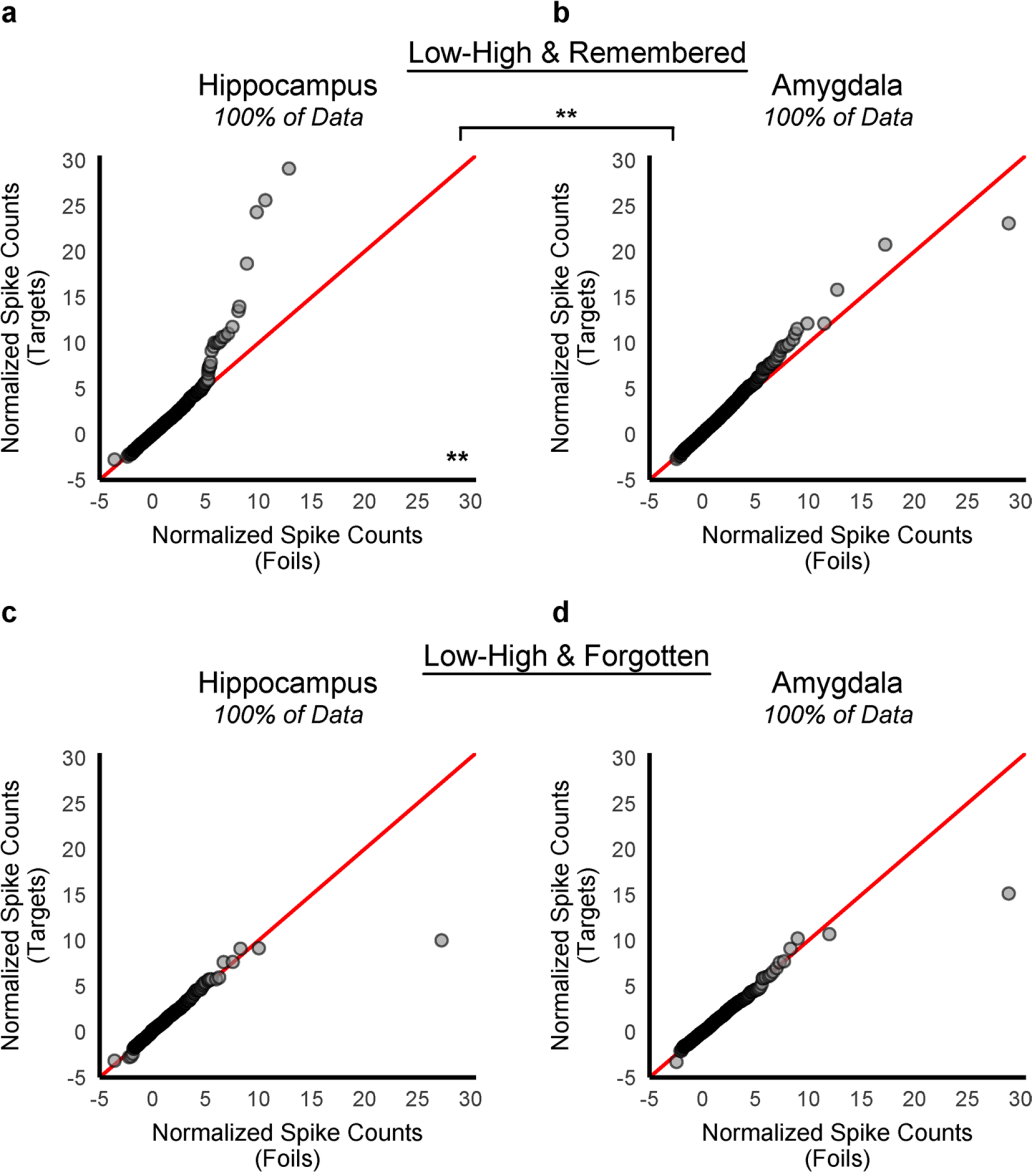
Empirical quantile–quantile (Q–Q) plots of the normalized spike count distributions at retrieval for targets with a relative increase in excitability at encoding as a function of subsequent memory. As in Figs. 1, 2 and 3, the distributions consisted of normalized spike counts from each neuron in response to each retrieval trial, across all patients and sessions. The most theoretically relevant and specific subset of targets associated with a relative increase in excitability during encoding (Low-High: changed from low spiking pre-stimulus onset to high spiking post-stimulus onset during encoding) was further divided into remembered and forgotten categories in the hippocampus (**a**: Low-High remembered, 41,340; **c**: Low-High forgotten, 38,447 recordings) and the amygdala (**b**: Low-High remembered, 56,840; **d**: Low-High forgotten, 52,673 recordings). In panel (**a**), a sharp deflection toward the y-axis was evident in the hippocampus for remembered targets that were associated with a relative increase in firing rate at encoding. In contrast, in the amygdala (**b**), most points fell densely on the diagonal line. Additionally, no other subset of targets split by other levels of spiking at encoding (Low–Low, High–High, High–Low) for remembered or forgotten items showed visual evidence of a difference in skewness compared to foils (Supplementary Fig. S5). The deflection observed in panel (**a**) disappeared after removing the top 0.25% of data, indicating that relatively few neurons fired strongly in response to remembered targets with a relative increase in firing rate at encoding compared to foil items, and only within the hippocampus (Supplementary Fig. S6). Thus, the difference in skewness associated with the item-specific memory signal was statistically reliable and selective to only the hippocampus, only to targets that were remembered, and only to targets associated with a relative increase in excitability at encoding (B = 10,000, *p* < 0.0125, two-tailed). **p < 0.01.

Indeed, as might be expected, the skewness difference (theoretically reflective of sparse coding) was selectively identified on the Q-Q plots of the hippocampal spike counts and for targets that were both (1) associated with relatively higher firing rates during encoding (Low-High) and (2) were also subsequently remembered (Fig. 4a). If the targets that were associated with relatively higher excitability during encoding were subsequently forgotten, no such effect was evident (Fig. 4c). This pattern also did not emerge in the amygdala for targets that exhibited relatively higher firing rates (Low-High) at encoding, whether those targets were later remembered (Fig. 4b) or forgotten (Fig. 4d). Additionally, no other encoding level-by-subsequent memory combinations within the hippocampus or amygdala (Supplementary Fig. S5) exhibited the skewness difference that we hypothesize arises from sparse coding.

**Fig. 5:**
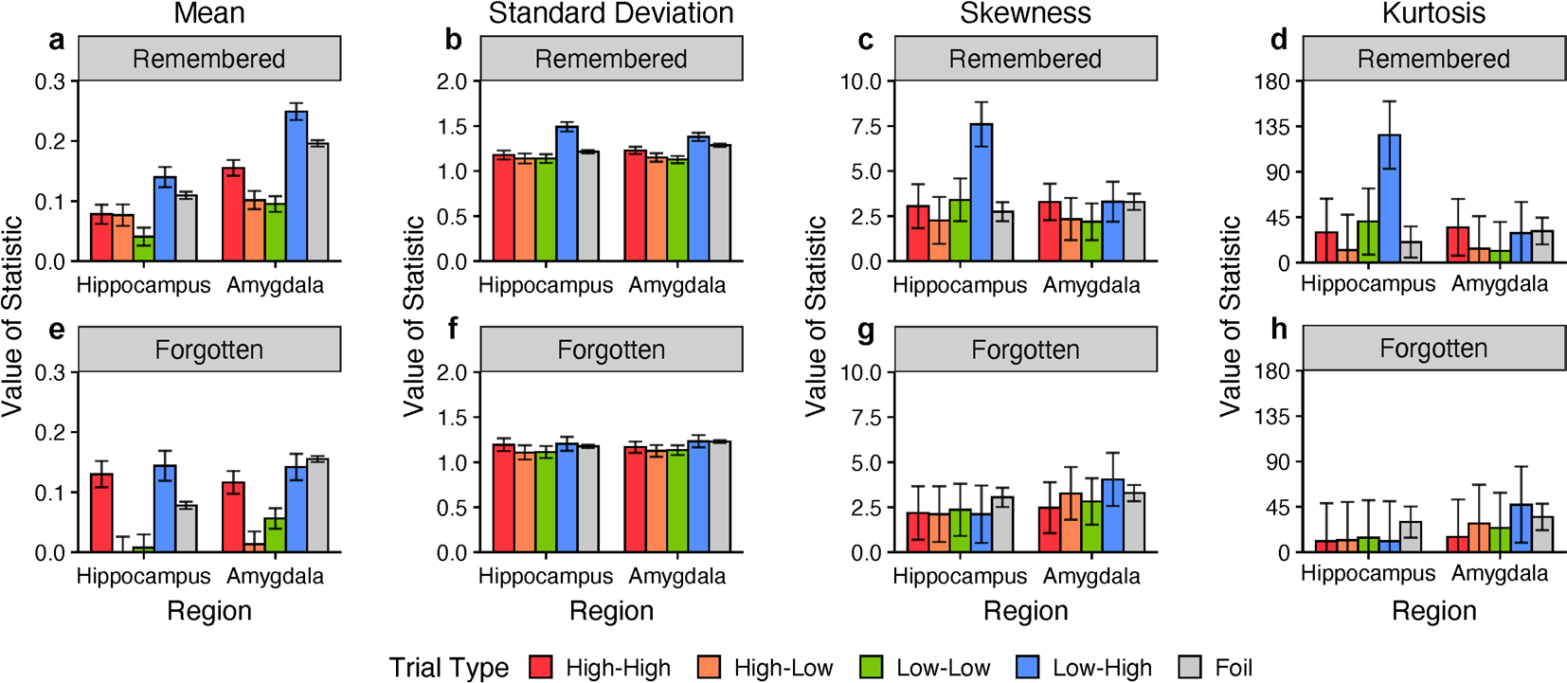
Statistical moments of the item-by-neuron normalized spike count distributions as a function of the pattern of relative excitability at encoding, subsequent memory, and region. Each bar chart represents one statistical moment of the normalized spike counts at retrieval for foils (gray) and targets, categorized by 1) the pattern of firing before and during item presentation at encoding (red: consistently high spiking [High–High]; orange: decrease in spiking [High–Low]; green: consistently low spiking [Low–Low]; blue: increase in spiking [Low–High]) and 2) subsequent memory response (remembered items: top row, forgotten items: bottom row). The primary statistical moment suggestive of sparse coding, skewness (**c**), was conspicuously elevated for the hippocampal spike counts in response to targets with relatively increased excitability at encoding (changed from low spiking pre-stimulus onset to high spiking post-stimulus onset firing during encoding, [Low-High: blue]) for remembered (top) but not forgotten (bottom) items, when compared to all other bars. The same pattern was also observed for the standard deviation (c) and kurtosis (d) values.,

The remarkable specificity of these findings is best appreciated by examining a bar graph (Fig. 5) of the four statistical moments of the response distributions (mean, standard deviation, skewness, and kurtosis) broken down by region (hippocampus and amygdala), subsequent memory, and the patterns of excitability at encoding. Each column plots one statistical moment (arrayed from left to right), with remembered targets shown in the top row, (Fig. 5a-d), forgotten targets shown in the bottom row (Fig. 5e-h), and each pattern of excitability at encoding is color-coded: (High-High: red, High-Low: orange, Low-Low: green, and Low-High: blue). The moments for the foil-by-neuron response distribution are shown in gray. For the foil items, the value for a given moment (e.g., the mean) was the same for the remembered-target graphs on top and the forgotten-target graphs on the bottom (i.e., the remembered vs. forgotten distinction does not apply to foil items). Within the comparison of skewness values, the top leftmost blue bar, representing the hippocampal recordings from retrieval in response to remembered targets with relatively higher firing rates at encoding (Low-High), conspicuously stands out among all other bars (Fig. 5c). A similar pattern was found for kurtosis (Fig. 5c), and to a lesser extent, for standard deviation (Fig. 5b).

In summary, the most specific and theoretically relevant subset of targets, items that were later remembered and were also associated with relatively higher firing rates at encoding, were the only targets that showed evidence of sparse coding. Moreover, this effect was evident only in the hippocampus.

## Discussion

A central challenge for memory research is to explain how individual episodic experiences are encoded in a form that is both specific and durable. Neurocomputational models propose that the hippocampus solves this problem by representing each event within sparse, pattern-separated neural assemblies^13–16^, thereby reducing interference between memories and supporting retrieval of their unique spatial and temporal context. Recent theories further suggest that the allocation of neurons to such assemblies is not random but reflects biases in neuronal excitability (intrinsic and learning-induced) and synaptic influences^30,38^. To test whether these principles extend to humans, we analyzed a publicly available dataset of single-unit recordings from the medial temporal lobe (MTL) of epilepsy patients performing an old/new recognition task^42,43^.

We replicated prior findings of item-specific, pattern-separated, sparse coding localized to the hippocampus^23–25^ (Fig. 1; Table 1) and extended them by identifying two theoretically distinct subsets of targets that exhibited evidence of a sparse coding scheme. Specifically, the sparse coding signal was present only for targets that elicited a relative increase in firing rate during encoding (Low-High: Fig. 2; Table 2), and for targets that were subsequently remembered (Fig. 3; Table 3). The sparse coding signal in the hippocampus further emerged only when evaluating the conjunction of targets that were categorized as Low-High at encoding and later remembered (Figs. 4 and 5c: Low-High, blue bars). In contrast, no other subsets of targets exhibited a sparse coding signal in the hippocampus, and there was no evidence of sparse coding within the amygdala (Fig. 5).

The specificity of our findings to the hippocampus is consistent with neurocomputational models of episodic memory representation. Sparse coding is thought to rely on two complementary hippocampal processes: pattern separation during encoding and pattern completion at retrieval. Although the recordings we analyzed here were not subregion-specific, pattern separation is thought to occur primarily in the dentate gyrus, while pattern completion is associated with CA3 ^26,27,44–46^. The functional specialization of these hippocampal subregions supports the view that sparse, item-specific representations formed during encoding are reinstated at retrieval.

In addition to identifying the sparse coding signal, a key mechanistic question is why specific neurons are allocated to a sparse code during learning. Rodent studies demonstrate that this process is biased rather than random, with intrinsic excitability, learning-induced changes, and synaptic factors influencing the recruitment of neurons into an engram^32,37,38^. For example, elevated CREB expression enhances intrinsic excitability and increases the probability of recruitment, as shown in the nucleus accumbens^47^ and amygdala of rodents^22^, and in CA1 of the hippocampus in rabbits^48^. Early-gene and CREB manipulation studies demonstrate that neurons with experimentally increased excitability are preferentially incorporated into memory traces compared to those with relatively reduced excitability^35^. Within this framework, neurons with low excitability (e.g., low CREB, potentially analogous to Low–Low in our data) would be less likely to be recruited, whereas neurons with high excitability (e.g., high CREB, potentially analogous to High–High) would be more likely to be recruited^29,30^. In our dataset, the sparse coding signal was not detected at retrieval for items with consistently low firing during encoding, consistent with this model, but it was also absent for items with consistently high firing. Together, these findings suggest that our results cannot be fully explained by changes in intrinsic excitability, as measured in rodents by CREB expression.

Instead, we found that the sparse coding signal emerged only in the hippocampus for individual targets that elicited a relative increase in firing at encoding (low firing pre-stimulus onset to high firing during stimulus onset) and that were later remembered (Low-High: Fig. 5c, blue bars). Prior work in animal models shows that learning can enhance intrinsic excitability of single CA1 neurons that share a temporal context during encoding^33,36^, and that transient optogenetic stimulation of amygdala neurons during learning was sufficient to bias those neurons toward allocation^49^. Together, this evidence suggests that learning-induced changes in excitability, not only pre-learning excitability levels, can bias allocation. Consistent with this interpretation, in our study, only items with a transient increase in firing of hippocampal neurons at encoding (Low-High), exhibited evidence of the sparse coding signal at retrieval (Table 2; Figs. 2a, 4 and 5c: blue bars), whereas sustained baseline firing (High–High: Table 2; Figs. 2c and 5c: red bars) did not. Thus, only transient firing at encoding predicted sparse coding at retrieval. This suggests that in our human recordings, allocation into a sparse code is influenced by learning-related changes during encoding rather than by pre-existing excitability alone, a pattern that is broadly consistent with animal models showing that both pre-existing and learning-induced excitability can bias neuronal allocation.

Our approach offers a novel perspective compared to rodent studies of gene expression by operationalizing excitability in terms of relative firing around stimulus onset (1s pre- vs. 1s post-onset). This high temporal resolution contrasts with prior rodent studies relying on early gene markers of activity measured over minutes, hours, and days. Although we cannot directly distinguish whether changes in firing rate in our data reflect a pre-existing neuronal excitability or learning-induced excitability, the observed Low–High firing pattern for remembered items seems most consistent with the learning-induced interpretation. Additionally, synaptic factors such as the connectivity, prior plasticity, momentary input may also modulate a neuron’s bias to fire^32,37,38^. Therefore, using firing rate as a proxy for excitability cannot distinguish between these influences and their contributions to spiking activity. Our findings are consistent with the idea that learning can influence a neuron’s bias to fire and thereby its allocation to a sparse code. By analyzing single-neuron firing with a temporally precise, stimulus-locked measure, we extend these principles to humans.

An alternative perspective has challenged the idea that humans represent episodic memories through a sparse coding scheme, unlike rodents and nonhuman primates^28^. The findings reported here serve as a rebuttal to this claim. Moreover, some of the work that gave rise to the idea that in humans, newly formed episodic memories are encoded via overlapping neural assemblies^50^ does not actually support that perspective (see Supplementary Material for full analysis). Additionally, some evidence of ensemble overlap in the CA1 of rodents has been observed during learning-induced changes with items learned closer in time^36^, suggesting that sparse codes may not be entirely orthogonal to each other, an effect that cannot be ruled out in our results. Finally, related work has identified “episode-specific” neurons in the human hippocampus that encode combinations of elements within an individual episode^51^, which could contribute to overlapping assemblies, thus challenging a strict sparse coding account.

There are several well-known limitations to intracranial recording techniques to evaluate cognition in patients with treatment-resistant epilepsy. The dataset included a small clinical sample size (*N* = 34), variable electrode placement, and the possibility of recording from diseased tissue. However, the dataset used here was released with well-established spike sorting and quality control procedures to minimize these sources of variability. Additionally, factors such as medication, sleep, or mood may have influenced cognition. Despite these limitations, single-unit recordings provide a rare opportunity to examine highly specific hypotheses of neural representations with precise spatial and temporal resolution.

The episodic memory signal we identified was item-specific, not generic. Although the generic signal has often been observed in the hippocampus, amygdala, and neocortex^23,39–41^, neurocomputational models have long proposed the item-specific memory signal is the fundamental hallmark of episodic memory. Consistent with long-standing neurocomputational models, our findings support the idea that individual episodic memories are represented by sparse, pattern-separated codes in the human hippocampus. Critically, this signal was observed only for targets that elicited a relative increase in firing at encoding and were subsequently remembered. This pattern is broadly consistent with the idea that learning-induced changes in excitability bias the recruitment of neurons into a memory trace and helps to bridge principles of neuronal allocation from animal models to sparse coding of episodic memories in humans.

## Methods

We analyzed an independent dataset of single-unit neural recordings from the medial temporal lobe of epilepsy patients during an old/new recognition memory task using visual memoranda. The dataset was originally made available by^43^ and was updated with additional participants by ^42^ in an OSF data repository (https://osf.io/hv7ja/). The dataset included participant characteristics, trial-by-trial paradigm information (e.g., stimulus presentation, timing), behavioral responses (memory judgment, confidence, and response time), and processed spiking data (i.e., localized electrodes, clustering, and raw signal filtering). Details of the experiment are described fully in previous work^40,41^ and the OSF repository and are summarized in the subsequent sections. In brief, we normalized the preprocessed raw spike counts from the published dataset, in combination with the trial-by-trial paradigm information and behavioral responses, to carry out our planned analyses.

### Independent dataset: human single-unit recordings from the medial temporal lobe

#### Participants

Patients with intractable epilepsy underwent depth electrode monitoring in preparation for potential surgical resection of the seizure loci. All participants were volunteers and provided written informed consent. In the OSF data repository, there were 87 available sessions collected across 59 participants (µ_age_ = 37 years; female = 25, male = 34). The experimental protocols associated with this dataset were approved by the Institutional Review Boards of the California Institute of Technology, the Huntington Memorial Hospital, and Cedars-Sinai Medical Center.

#### Single-unit data acquisition and processing

The independent dataset published preprocessed spike counts from macro-micro depth electrodes localized to the human hippocampus and amygdala for each experimental session. Each electrode consisted of eight 40 am diameter microwires, and electrodes were referenced to a ninth low impedance wire (Ad-Tech Medical). Electrodes were localized using MNI coordinates defined by post-operative MRI scans which were registered to the MNI152-aligned CIT168 template brain35 atlas. Broadband recordings (0.1–9,000 Hz filter) were sampled at 32 kHz using a Neuralgy Inc. system (ATLAS or Cheetah). Continuous recordings of the raw signal were bandpass filtered at 300–3,000 Hz for each electrode. Spikes were detected using local energy thresholds and were sorted using OSort^52^, which was used to identify clusters that represented putative single neurons. For each cluster, the following criteria were evaluated to determine individual units: (1) the shape of the mean waveform, (2) the interspike interval distribution, (3) violation of the refractory period (< 3% of the spikes have an ISI of less than 3 ms), and (4) stable firing rate and waveform amplitude during the task. Spike sorting quality was checked by quantifying the isolation distance, mean waveform, and signal-to-noise ratio.

#### Recognition memory task

Intracranial recordings were acquired while patients completed a recognition memory task with distinct encoding and retrieval phases (Supplementary Fig. S7). The independent dataset reported the trial-by-trial paradigm information and behavioral responses for each recording session. During the encoding phase, participants studied 100 images from five different visual categories: animals, people, cars/vehicles, outdoor scenes/houses, and flowers/food items. They were instructed to carefully study the images as their memory would be tested later. Each learning trial consisted of a 1-second delay (blank screen), a 1- or 2-second presentation of an image, another 0.5-second delay (blank screen), and a yes/no encoding question (i.e., “Is this an animal?”), with unlimited time to respond. At encoding, participants studied each image once except during 4 sessions in which the experimenters presented patients only 50 images due to poor performance during the encoding question. The results do not change appreciably whether or not the data from these 4 sessions are included.

After the encoding phase, participants completed a distractor task (approximately 15 min) to inhibit active rehearsal. They then completed an old/new recognition memory test with confidence ratings. During the retrieval phase, 100 images were shown; 50 were previously shown during the learning phase (“old”; targets) and 50 were not presented before during learning (“new”; foils). Patients used a button box to respond on a 1–6 confidence scale (1 = new, very sure; 2 = new, sure; 3 = new, guess; 4 = old, guess; 5 = old, sure; 6 = old, very sure). Each recognition trial consisted of a 1-second delay (blank screen), a 1- or 2- second presentation of a target or foil item, another 0.5-second delay (blank screen), and were then prompted to submit a confidence rating (i.e., 1–6) with unlimited time to respond.

### Current report: investigation of sparse coding at retrieval based on firing at encoding and subsequent memory

This report investigates the predictions of neurocomputational models of sparse coding in the human hippocampus, using data from a freely available single-unit recording dataset collected during an old/new recognition memory task (described above). We specifically tested for evidence of sparse coding within single-unit recordings of the hippocampus by comparing the shapes of the target item and foil item spiking distributions of the hippocampus and amygdala and tested whether those differences were statistically reliable. Our statistical approach to detecting sparse coding is tailored and non-standard, and it likely would not be applied to single unit recordings absent the predictions of the neurocomputational models we seek to test.

#### Statistical method to detect the sparse coding signal

For each neuron, and for each item presented during both the learning (encoding) phase and the recognition (retrieval) phase, we calculated the spike counts that occurred during the pre-stimulus onset period (-1000ms to 200ms) and the post-stimulus onset period (200ms to 1000ms), to obtain measures of single neuron activity before and during the presentation of each item. In addition to examining raw spike counts during encoding, for the retrieval analyses, the recordings were normalized to account for differences in baseline firing rates across neurons. Doing so yielded normalized spike counts in response to each item (*i*) during the recognition test, separately for each neuron (*j*). The spike counts from the pre-stimulus onset period across all learning and recognition trials within that session were considered as the baseline firing for each neuron (*j*), yielding the pre-stimulus mean (µ_prestim*j*_) and standard deviation (σ_prestim*j*_) per neuron. Next, normalized post-stimulus onset spike counts were computed for every neuron’s response during the presentation of every test image (N_norm*ij*_) using the following equation: N_norm*ij*_ = (N_poststim*ij*_ - µ_prestim*j*_)/σ_prestim*j*_, where N_poststim*ij*_ represents the number of spike counts recorded for neuron *j* during the presentation of item *i*.

In contrast to the sparse signal we investigated, the more commonly investigated generic recognition memory signal is, by definition, not item specific and instead consists of a statistically significant difference in the average firing rate to old items compared to new items on the recognition test (see relevant analyses in Supplementary Material). Our focus here is on detecting a sparsely coded episodic memory signal, if it exists. A sparse episodic memory signal consists of a small fraction of neurons responding strongly to a small fraction of “old” items. Thus, a different approach is needed to detect this signal. Critically, testing for a neuron’s signal associated with a single old item can be performed only once. Testing the same neuron’s response to the same item multiple times would create new episodic memories of the test itself, which might influence each subsequent test. Multiple testing of neuron-item pairs might also simply identify a concept neuron (i.e., a semantic memory signal). These considerations are why we adopted the analytical approach described next.

A single neuron exhibiting an item-specific response on a single test would not necessarily reflect a sparse episodic memory signal for that old item. For example, it might instead reflect a semantic memory signal, one that would have occurred even if the word were new, or it might simply reflect an artifactual noise signal that happened to coincide with the presentation of the test item. However, both semantic memory signals and artifactual noise signals would be expected to occur with equal probability to old items (targets) and new items (foils). Therefore, the prediction of interest applies to a comparison of the full distributions of item-by-neuron spike counts for targets vs. foils.

Consider a simplified concrete example, illustrated in Supplementary Fig. S8, in which each recording represents the response of one neuron to one specific item. In this example, simulated recordings were measured from 100 single neurons during the presentation of 100 items on an old/new recognition test (50 old items and 50 new items). The two distributions of interest would consist of the 5,000 target-by-neuron responses to old items (100 neurons X 50 old items) and the 5,000 foil-by-neuron responses to new items (100 neurons X 50 new items). In one scenario, the target-by-neuron (mean = 0.78 and SD = 0.98) and the foil-by-neuron (mean = 0 and SD = 1.02) response distributions share a similar unimodal shape but differ in mean and SD. The probability density function of the target distribution would be shifted toward the right compared to the foil distribution. This would represent an overall higher mean firing rate for target items compared to foil items, and not the item-specific memory signal.

In another scenario, the same foil-by-neuron response distribution (mean = 0, SD = 1.02; 5,000 recordings) is compared to a bimodal target distribution that hypothetically reflects the presence of the sparse coding signal. The bimodal distribution exhibits a greater skewness compared to the foil distribution, due to a small proportion of recordings contributing to the upper mode (mean = 5, SD = 1) of the target distribution. The lower mode, which contains the majority of target-by-neuron responses, is similar in shape (mean = -0.4, SD = 1) to the foil-by-neuron response distribution. This similarity indicates that the neurons are responding to *individual* items approximately the same for both foils and targets. In a strict sparse coding scheme, a singular neuron would respond strongly to only one target item, but not to any of the other target items, nor to any of the foil items. The upper mode reflects a small number of strong responses to few target items, theoretically indicative of sparse coding. Statistically, this manifests as an increased positive skew in the target-by-item response distribution compared to the foil-by-item response distribution. Moreover, this difference in skewness should also reflect the influence of a very small percentage of target-by-neuron responses.

In reality, the underlying target and foil neuron-by-response distributions would not be perfectly Gaussian. Nonetheless, the principle that the target item distribution should be more positively skewed compared to the foil distribution still holds: only a small percentage of the target-by-neuron responses contribute to that skewness, and the remaining percentage of target-by-neuron recordings demonstrate the same shape as the foil-by-neuron distribution. Supplementary Fig. S8 illustrates the sparse coding signature by visually comparing the relative shapes of the hypothetical target distributions (unimodal and bimodal) to the unimodal foil distribution using empirical quantile-quantile (Q-Q plots). In an empirical Q-Q plot, the recordings for the foils and targets are rank-ordered separately and plotted on separate axes to visually compare the shapes of the distributions. A non-intuitive aspect of this approach is that the x-value for a given point on the plot (normalized spike count of a neuron in response to a particular foil) and the y-value for that same point (normalized spike count of a neuron in response to a particular test target) do not necessarily represent responses of the same neuron. A linear pattern indicates that the two distributions share the same or similar overall shape, even if the means or standard deviations differ (i.e., even if they differ in the first two moments). If the points are linear but shifted away from the red line of equivalence toward the y-axis, this pattern reflects that there is overall greater mean firing for the targets compared to the foils, which is not indicative of the sparse coding signal.

Conversely, nonlinear patterns in an empirical Q-Q plot reveal differences in skewness (the third moment) or kurtosis (the fourth moment) between the two distributions. For instance, Supplementary Fig. S8 depicts a difference in skewness between the hypothetical bimodal target and unimodal foil distributions, wherein most of the points fall on the linear red line of equivalence, but with a non-linear deflection at the upper quantiles toward the target distribution (y-axis). This pattern (linear except a deflection toward the y-axis at upper quantiles), indicates most recordings from both the target and foil distributions share a similar shape, but a small proportion of high firing recordings in response to few target items, consistent with a sparse coding scheme. This deflection can also be statistically confirmed if the shape of the target item distribution has increased skewness compared to the foil item distribution.

The nonlinear Q-Q plot pattern would be observed even if only a small percentage of target recordings exhibit differentially elevated firing (though the visual appearance of the plot might suggest otherwise). To test whether this pattern reflects sparse coding, a small proportion of the highest firing recordings (e.g., 0.25%) can be removed from both the target and foil item distributions. In Supplementary Fig. S8, the deflection disappeared when removing the top 0.25% of data from both distributions (bimodal target vs. unimodal foil), demonstrating that the deflection in these hypothetical data was driven by only a small proportion of high firing neurons in response to target items. In contrast, when the same approach is applied to the shifted pattern (unimodal target vs. unimodal foil), the pattern remains unchanged and reflects that the target items elicited a higher response at retrieval compared to novel items, most akin to the generic memory signal.

To identify the presence of the sparse coding signal in the empirical data we analyzed, Q-Q plots were generated using the methods summarized above and in Supplementary Fig. S8. The normalized target-by-neuron and foil-by-neuron response distributions were compared across all subjects and sessions, which included the spike count of each neuron’s response to every trial. We compared, both visually and statistically, the shapes of the target-by-neuron and foil-by-neuron normalized spike count distributions for each medial temporal lobe region separately (hippocampus: 73,539 item-by-neuron recordings from 736 neurons and amygdala: 99,944 item-by-neuron recordings from 1,038 neurons). To compare them visually, empirical quantile-quantile (Q-Q) plots were generated. The goal of this method is to visually assess whether two separate distributions have the same or similar shapes.

Statistically, the moments associated with the two empirical distributions were compared using bootstrap analyses. The empirical data consisted of N_t_ target-by-neuron normalized spike counts and N_f_ foil-by-neuron normalized spike counts across all subjects and all neurons. If the Q-Q plots of our empirical data match the hypothetical pattern in Supplementary Fig. S8, we predict that skewness values of the normalized spike count distribution would be higher for targets compared to foils, specifically in the hippocampus and not the amygdala. The sparse coding signal would be evident as an interaction effect between the two MTL regions of interest (hippocampus and amygdala) and the skewness of the two distributions (target and foils) in which the difference in skewness between the target and foil distributions is greater in the hippocampus relative to the corresponding difference in the amygdala. First, for each statistical moment (i.e., mean, standard deviation, skewness, and kurtosis), the empirical interaction value was calculated (e.g., the difference in the observed skewness values between the target and foil distributions for the amygdala was subtracted from the corresponding difference for the hippocampus).

Next, we combined the N_t_ and N_f_ empirical recordings to conduct the bootstrapping procedure. For each of B *=* 10,000 bootstrap trials, a bootstrapped “target” distribution of N_t_ observations was created by randomly sampling with replacement from the combined empirical data, and a bootstrapped “foil” distribution of N_f_ observations was created by randomly sampling with replacement from the combined empirical data. We then calculated a difference score representing the difference in the relevant statistical measure (e.g., skewness) between these two randomly sampled distributions. Repeating this process for B *=* 10,000 bootstrap trials yielded 10,000 bootstrap difference scores for the relevant statistic. The proportion of bootstrapped trials for which the absolute value of the interaction difference score was greater than or equal to the absolute value of the empirically observed interaction difference score determined the p-value. Significance was defined as *p* < 0.05 (two-tailed), unless otherwise specified. The bootstrap procedure was repeated for the interaction difference score for each statistical moment separately. To determine the direction of the interaction effects, the same bootstrap procedure was used to examine if the difference in each statistical moment between the target and foil distributions was statistically reliable, within the hippocampus and the amygdala separately.

#### Partitioning episodic memories by encoding firing patterns

After performing this omnibus analysis, we investigated whether the sparse coding signature during retrieval, defined as a significant interaction between brain region (hippocampus vs. amygdala) and the target–foil skewness difference, was selectively associated with two specific circumstances that should theoretically give rise to it: (1) neurons with a relative increase in firing rate at encoding (i.e., relative increase in neuronal excitability) and (2) remembered episodic memories. This prediction is guided by allocation models in animals in which excitability influences neuronal recruitment into memory traces. First, we investigated whether the sparse coding signature was selectively present at retrieval for targets with relatively increased firing rates during encoding. Within each session, the mean pre-stimulus onset (-1000ms-200ms) and mean post-stimulus onset (200ms-1000ms) raw spike counts during the encoding phase (i.e., during list presentation) were calculated for each item. Items were then rank-ordered by the raw mean spike count value and placed into “Low” or “High” groups using a median split, first for pre-stimulus onset values and again for post-stimulus onset values. Items that were at the median value pre- or post-stimulus onset were excluded from the primary analysis. This resulted in target items being partitioned into one of four categories based on the level of firing pre- and post-stimulus onset presentation: (1) increase in firing: Low-High, (2) consistently high firing: High-High, (3) decrease in firing: High-Low, and (4) consistently low firing: Low-Low. Items most strongly associated with a relative increase in excitability were defined to be those in the Low-High category, and the question of interest was whether a difference in skewness between the target-by-neuron response distribution and foil-by-neuron response distribution was limited to those items.

Second, we investigated whether the sparse coding signature was selectively associated with items that were successfully remembered (i.e., correct “old” decisions on the recognition test). To address this issue, after classifying targets into one of four categories based on their responsivity during encoding (e.g., Low-Low), the targets were further classified within each category as remembered or forgotten. The question of interest was whether a difference in skewness between the target-by-neuron response distribution and foil-by-neuron response distribution would be limited not only to targets associated with neurons that relatively increased in excitability at encoding (Low-High) but also to a further subset of those targets that were successfully retrieved during the recognition test.

## Supplementary Information

Below is the link to the electronic supplementary material.

Supplementary Material 1

## Data Availability

The dataset analyzed during the current study is available in the following Open Science Framework repository: [https://osf.io/hv7ja/](https:/osf.io/hv7ja) .
