# Supplementary material for: Neuronal allocation and sparse coding of episodic memories in the human hippocampus
Source: Sci Rep. 2025 Nov 18;15:40430. doi: 10.1038/s41598-025-21967-7 (PMC12627520; doi:10.1038/s41598-025-21967-7)
Supplement: Supplementary file 1 — Supplementary Material 1 [file 41598_2025_21967_MOESM1_ESM.docx]

**Supplementary Figure 1. Empirical Q-Q plots of the normalized spike count distributions at retrieval partitioned by encoding firing patterns where sparse coding signal was not detected**

**
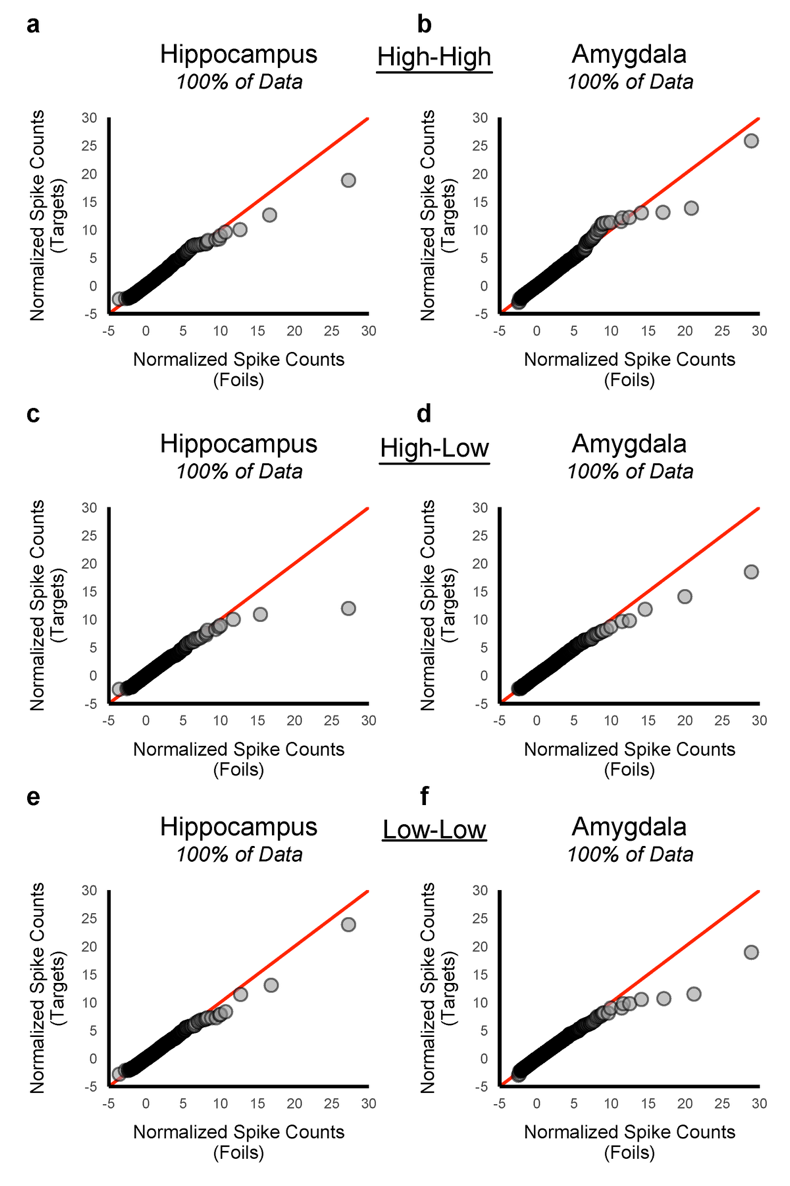
**

*Note*. Empirical quantile–quantile (Q–Q) plots of the normalized spike-count distributions at retrieval partitioned by relative excitability at encoding, where the sparse-coding signal was not detected. The distributions consisted of normalized spike counts at retrieval from each neuron’s response to each trial across all patients and sessions. Targets were divided into subsets based on relative excitability during encoding (spiking activity immediately before and during stimulus presentation): High–High (a,b), High–Low (c,d), Low–Low (e,f), and Low–High (Main Text Fig. 2). For each category, the foil-by-neuron response distribution (x-axis) was compared with the corresponding target-by-neuron response distribution in the hippocampus (a: High–High 44,821; c: High–Low 42,991; e: Low–Low 45,143 recordings) and amygdala (b: High–High 62,513; d: High–Low 59,334; f: Low–Low 63,108 recordings). No nonlinear deflection (i.e., no evidence of sparse coding) was detected in the hippocampus or amygdala for the High–High, High–Low, or Low–Low encoding patterns. Only targets with a relative increase in excitability at encoding (Low–High) showed the sparse-coding signal at retrieval, and only within the hippocampus. Refer to Main Text Table 2 and Supplementary Table 2 for detailed statistics. See Supplementary Figure 2 for the corresponding Q–Q plots after removing the top 0.25% of both distributions.

**Supplementary Figure 2. Empirical Q-Q plots of the normalized spike count distributions at retrieval partitioned by encoding firing patterns with the top percentage of data removed**

*Note*. Q–Q plots showing the shapes of the target-by-neuron and foil-by-neuron distributions partitioned by encoding firing pattern, with the top 0.25% of recordings removed from both distributions. Compare these plots with the 100% data plots in Main Text Fig. 2 (Low–High) and Supplementary Figure 1 (High–High, High–Low, Low–Low).For the hippocampus, most spike counts fell densely on the diagonal, with a sharp upward deflection toward the y-axis in Main Text Fig. 2a, indicating that Low–High targets had a more skewed distribution than foils. In panel (a), this deflection disappeared after removing the top 0.25% of data, showing that relatively few hippocampal neurons fired strongly to targets that had shown increased firing at encoding. No differences were observed for the amygdala at either 100% (Main Text Fig. 2b) or 99.75% (panel b) data. None of the Q–Q plots indicated skewness differences between target and foil distributions for any other encoding patterns under either 100% (Supplementary Figure 1) or 99.75% (panels c–h) data.

**Supplementary Table 1. Statistical results for mean, standard deviation, and kurtosis of the target vs. foil distributions at retrieval partitioned by encoding firing patterns**

*Note.* No significant interactions were present for a given statistical moment between the target-vs.-foil normalized spike count distributions in one region (e.g., the hippocampus) compared to the corresponding difference in the other region (e.g., the amygdala) according to bootstrap tests (*B*=10,000, Bonferroni corrected, p < 0.0125, two-tailed). Within the hippocampus, a significant difference between the target vs. foil mean (p < 0.001), standard deviation (p < 0.001), and kurtosis (p = 0.012) was observed only for targets that elicited an increase in firing rate at encoding (Low-High). Within the amygdala, a significant difference (p < 0.001) was present for the difference in means for the targets vs foils for those targets associated with an increase in firing at encoding (Low-High), decrease in firing (High-Low), and those that remained low firing (Low-Low). Additionally, within the amygdala, a significant difference was present for the difference in standard deviation for the targets vs foils for only those targets associated with a relative increase in firing at encoding (Low-High: p = 0.005) and standard deviation for the targets associated with low firing at encoding (Low-Low: p = 0.007). The corresponding analysis for skewness is reported in Table 2 of the main text. Bonferroni corrected p < 0.0125, * = p < 0.05, ** = p < 0.01, *** = p < 0.001

**Supplementary Figure 3. Empirical Q-Q plots for remembered targets vs. foils and forgotten targets vs. foils with the top percentage of data removed**

*Note*. Q–Q plots comparing spike-count distributions for remembered targets vs. foils (panels a,b) and forgotten targets vs. foils (panels c,d), with the top 0.25% of recordings removed from each distribution. Compare these plots with the 100% data plots in Main Text Figure 3. In the hippocampus, most spike counts fell along the diagonal, with a sharp upward deflection toward the y-axis in Main Text Fig. 3a, indicating greater positive skew for remembered targets than foils. In panel (a), this deflection disappeared after removing the top 0.25% of data, showing that only a small subset of neurons fired strongly to remembered targets. No deflection was seen for the amygdala or for forgotten items in either region.

**Supplementary Table 2. Analyses of the statistical moments of remembered target vs. forgotten target distributions at retrieval**

*Note.* A significant interaction reflects a greater difference in a statistical moment (e.g., skewness) of the spike count distributions for the remembered vs. forgotten targets in one region compared to the other region (hippocampus vs. amygdala) according to bootstrap tests (B *=*10,000, p *<* 0.05, two-tailed). The interactions were significant for the mean (p = 0*.*0015) and skewness (p = 0.034), and approached significance for kurtosis (p = 0*.*059). Within each region considered separately, differences in the upper moments of the distributions were detected in the hippocampus (skewness: p = 0*.*022, kurtosis: p = 0*.*0501) but not the amygdala (skewness: p = 0*.*732 and kurtosis: p = 0*.*789). Conversely, mean firing was greater for remembered compared to forgotten items in the amygdala (p *<* 0*.*001) but not in the hippocampus (p = 0*.*156). Although the interaction was not significant for standard deviation, within each region, both the hippocampus exhibited a significant difference (p = 0*.*020) and the amygdala approached significance (p = 0*.*055), with greater SD values for remembered compared to forgotten items. * = p < 0.05, ** = p *<* 0.01, *** = p *<* 0.001.

**Supplementary Figure 4. Empirical Q-Q plots for remembered vs. forgotten targets**


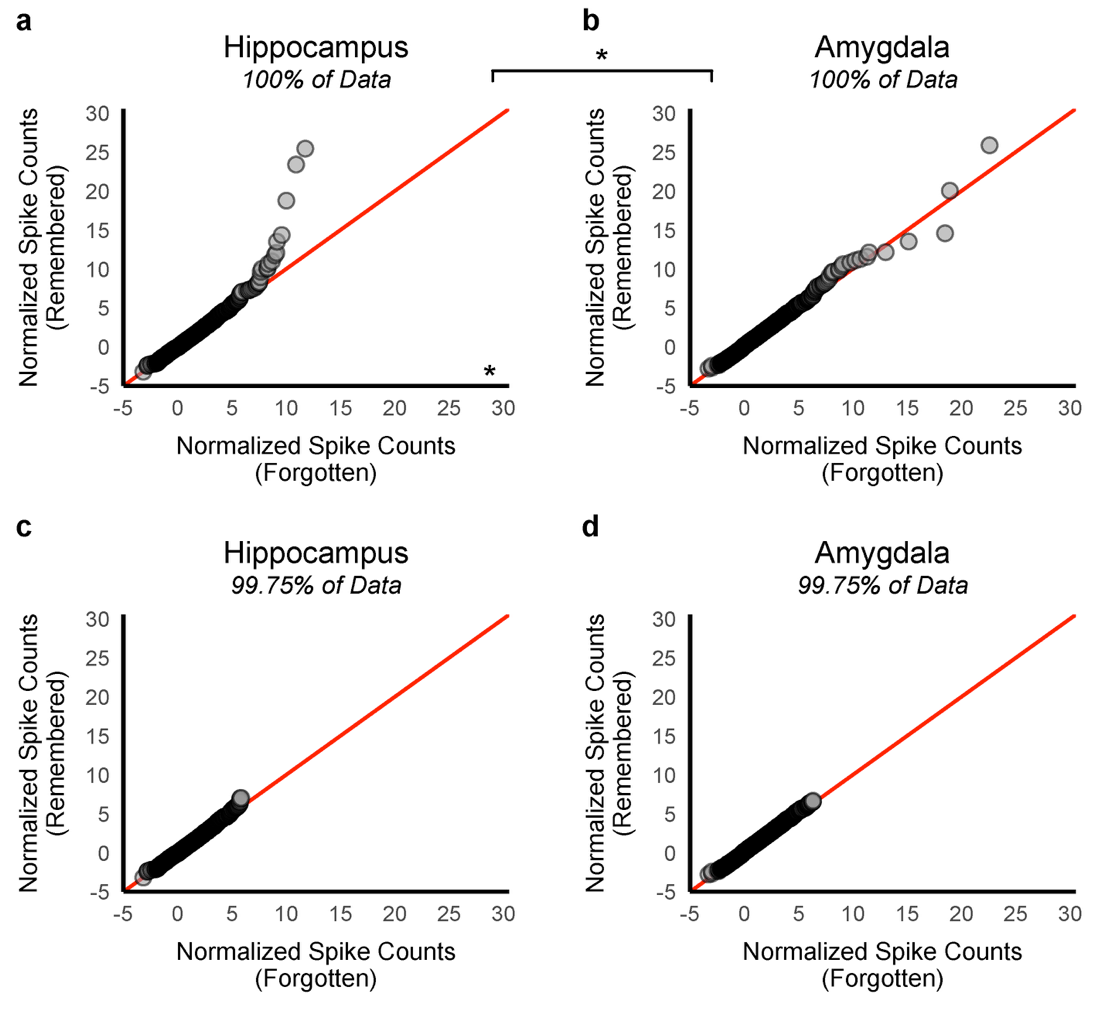


*Note*. Normalized spike-count distributions comparing remembered targets (y-axis) and forgotten targets (x-axis) for the (a) hippocampus and (b) amygdala. A sharp upward deflection was evident for the hippocampus but not the amygdala. As shown in Supplementary Table 2, the interaction between distribution skewness (remembered vs. forgotten) and region (hippocampus vs. amygdala; 36,298 vs. 50,092 recordings) was significant. Within each region, the hippocampus showed a significantly greater skew difference (remembered > forgotten), whereas the amygdala did not. In panel (c), the deflection disappeared after removing the top 0.25% of data, indicating that relatively few neurons fired strongly to remembered targets. * = p < 0.05.

**Supplementary Figure 5. Empirical Q-Q plots for targets partitioned by encoding firing patterns as a function of subsequent memory**

|  |  |
| --- | --- |
| **** |  |

*Note*. Q–Q plots showing remembered and forgotten target-by-neuron vs. foil-by-neuron distributions partitioned by encoding firing pattern. Panels are organized by encoding pattern (rows) and region (columns). Significant differences in skewness and visual evidence of the item-specific memory signal for the Low–High condition are reported in Main Text Fig. 4. No differences in skewness between target-by-neuron and foil-by-neuron distributions were detected in either region for remembered or forgotten items within the other encoding patterns (High–High a,b,e,f; High–Low c,d,g,h; Low–Low i–l).

**Supplementary Figure 6. Empirical Q-Q plots for targets partitioned by encoding firing patterns as a function of subsequent memory with the top percentage of data removed**

*Note*. Q–Q plots showing remembered and forgotten target-by-neuron vs. foil-by-neuron distributions partitioned by encoding firing pattern, with the top 0.25% of data removed. Panels are organized by encoding pattern (rows) and region (columns). In Fig. 4a of the main text, significant differences in skewness and visual evidence of the item-specific memory signal for the Low–High condition are reported. In panel (a) here, that deflection disappeared after removing the top 0.25% of data, showing that only a small subset of hippocampal neurons fired strongly to targets that were remembered and showed increased excitability (Low–High) at encoding. This effect was not present in the amygdala (Main Text Fig. 4b and panel b). No differences in skewness were detected in either region for remembered or forgotten items within the other encoding patterns under either the 100% (Supplementary Figure 5) or 99.75% (panels b–p) datasets.

## **Generic memory signal**

The generic recognition memory signal is (by definition) not item specific and instead consists of a difference in the average firing rate to old items compared to new items on the recognition test. This difference might be observed either at the level of single neurons (e.g., a neuron with a significantly higher firing rate to old items vs. new items) or at the population level (aggregated measurements of all recorded single neurons exhibiting a higher rate of responding to old items vs. new items). Bootstrapping analyses determined whether a higher firing rate for target or foil items was observed in the hippocampus or amygdala. For each single neuron, the bootstrap trials (B=10,000 trials) compared the mean normalized firing rate for target and foil items. Each bootstrap trial 1) combined the target (N_target_) and foil (N_foil_) item normalized spike counts 2) randomly sampled with replacement from that combined set of measurements to generate a new set of N_target_ normalized “target” spike counts and a new set of N_foil_ normalized “foil” spike counts, then 3) calculated the difference in the means between the two sampled distributions, resulting in 10,000 mean different scores from the bootstrapped samples. The proportion of these trials in which the absolute value of the difference in means between the “target” and “foil” distributions was greater than or equal to the observed difference in the original target and foil distributions determined the p value. Significance was defined as p < 0.05 (two-tailed), unless otherwise specified.

As defined above, a difference in neural activity in response to previously studied items (targets) or foil items (foil) items is what we refer to as the “generic” episodic memory signal. The generic signal can be measured at the level of individual neurons or at the population level. Individual neurons that exhibit this property (i.e., firing rates that differ for targets vs. foil) are considered to be memory-selective. Memory-selective neurons that increase in spike count in response to targets compared to foil items are “repetition detectors”, and those that increase in spike count in response to foil items compared to targets are “novelty detectors”. The generic memory signal in single neurons has been observed in prior work when examining all responses irrespective of the behavioral response (Urgolites et al., 2022) or when excluding error trials (Rutishauser et al., 2010, 2015). We identified the generic memory signal in single neurons in the hippocampus and in the population firing of the amygdala.

Each recorded neuron was tested for the generic memory signal across all patients and sessions. The test compared spikes for targets vs. foil items during the poststimulus window of 200ms to 1-second after the stimulus presentation (see Methods; replication of Urgolites et al., 2022). Using this poststimulus window for all trials irrespective of behavioral response, the generic signal was not detected. More specifically, of the 736 hippocampal neurons, 32 (4.3%) were memory-selective, a proportion not significantly greater than the 36.8 expected by chance (α = 0.05; 736 × 0.05 = 36.8). Among these, 18 neurons were repetition detectors, and 14 neurons were novelty detectors. Of the 1043 amygdala neurons, 50 (3.8%) were memory-selective, not significantly exceeding the 52.2 neurons expected by chance (α = 0.05; 1038 × 0.05 = 51.9). Among these, 21 neurons were repetition detectors, and 29 neurons were novelty detectors.

Next, we aimed to replicate the identification of the generic memory signal previously reported with a subset of this dataset (Rutishauser et al., 2015). Following a similar approach, we extended our analysis to include spike counts within an extended poststimulus window of 200ms to 1.7-seconds after image onset, while also excluding error trials. Using this method, the generic memory signal was detected in 58 of the 736 hippocampal neurons (7.9%), significantly exceeding the number of neurons expected at chance (α = 0.05; p = 0.0005). Among these neurons, 34 were repetition detectors and 24 were novelty detectors. Of 1038 amygdala neurons, 50 (5.5%), were memory-selective, which was not significantly greater than chance. Of these, there were 23 repetition detectors and 27 novelty detectors. Therefore, we replicated the identification of the generic memory signal when examining correct responses during an extended post-stimulus window and found it was selective to the hippocampus.

Additionally, the difference in normalized response count for target and foil items in the hippocampus and amygdala was tested across all patients and sessions, for both poststimulus windows and trial types of the single neuron analyses of the generic memory signal. For the replication of Urgolites et al. (2022), mean firing in the amygdala was significantly greater for foil compared to target items (B = 10,000, two-tailed, p = 0.0374, mean target = 0.14,mean foil = 0.16; reported as one-tailed in Table 1). No significant difference in population firing was detected in the hippocampus between target and foil items (B = 10,000, two-tailed, p = 0.667, mean target = 0.08, mean foil = 0.08; reported as one-tailed in Table 1). For the replication of Rutishauser et al. (2015), mean firing in the amygdala was significantly greater for foil compared to target items (B = 10,000, two-tailed, p = 0.0027, mean hit = 0.15, mean correct rejection = 0.18). No significant difference in population firing was detected in the hippocampus between target and foil items (B = 10,000, two-tailed, p = 0.314, mean hit = 0.07, mean correct rejection = 0.06). We identified the generic memory signal in single neurons in the hippocampus and in the population firing of the amygdala

## **Evidence against the notion newly formed episodic memories are encoded via overlapping neural assemblies**

The idea that episodic memories are represented by overlapping (not pattern-separated) neural assemblies was based on work involving “concept cells” (Quiroga, 2012). As noted by Quiroga (2020), concept cells fire to a particular concept, such as a famous person (e.g., James Brolin) and not to other concepts. A concept cell is activated whether participants are looking at pictures of the person, reading the person’s name, or “…even when recalling or thinking about the person” (p. 1002). In short, any stimulus that triggers the thought of the famous person activates the concept cell. Concept cells reflect semantic memory, but their apparent role in episodic memory has been taken as evidence against pattern separation. For example, if the “preferred” (p) concept like James Brolin is experimentally associated with a different “non-preferred” (Np) stimulus like the Eiffel Tower in a single-trial episodic memory task (e.g., a picture of James Brolin standing next to the Eiffel Tower), some concept neurons immediately expand their tuning in such a way as to now also fire to the Np stimulus (Ison et al., 2015). Critically, immediately upon learning the association, the neuron continued responding to Brolin and now also fired to Brolin standing near the Eiffel Tower and to the Eiffel Tower without Brolin. These neurons were referred to as “pair-coding” neurons. The existence of these neurons was interpreted as being inconsistent with the notion that episodic memories are coded in distinct, pattern-separated assembles because the episodically learned associations were encoded by expanding the tuning of the neurons initially responding to a concept, not by recruiting new neurons to form non-overlapping (pattern-separated) representations.

However, Ison et al. (2015) pointed out another possible interpretation of their findings that did not require the assumption that the concept neuron episodically expanded its tuning at all. As they put it: “Two possible mechanisms can in principle account for the increased response to the Np stimuli after learning. On the one hand, neurons can rapidly change their tuning and start firing to the Np stimuli directly—that means, a neuron originally encoding the p stimulus starts encoding the Np stimulus after learning—in which case, the time courses of both p and Np signals are expected to be similar. On the other hand, the Np stimuli can act as a cue to evoke the representation of (and in turn the neuron’s firing to) the p stimuli” (p. 226). According to the second interpretation, the James Brolin concept neuron responded to non-preferred Eiffel Tower stimulus after associative learning only because it now triggered the thought of James Brolin. As noted above, a concept neuron is defined as a neuron that responds whenever any stimulus triggers a thought of its preferred concept. According to this interpretation, the fact that the James Brolin neuron now responds to the Eiffel Tower only means that it is still behaving as a concept neuron (not that it has expanded its turning based on an episodically learned association).

To distinguish between these two possible mechanisms, Ison et al. (2015) analyzed the response onset latencies of the pair-coding neurons to the p and Np stimuli. If the concept neuron expanded its tuning based on the newly learned episodic association, similar latency onsets for the p and Np stimuli should be observed. If the concept neuron did not expand its tuning but is instead activated by the Np stimulus because it triggers the thought of the p stimulus, a longer latency onset should be observed for the Np stimulus compared to the p stimulus. Out of 21 pair-coding neurons, 13 showed no significant difference in latency between the p and Np stimuli (these were labeled Type I neurons), and 8 showed a significantly slower latency for Np stimuli compared to p stimuli (these were labeled Type II neurons), as if the Np stimuli merely triggered the thought of their corresponding p stimuli. The existence of Type I neurons was taken as evidence that some neurons did indeed expand their tuning as a result of the episodically learned association between the p and Np stimuli. This finding was interpreted as being inconsistent with pattern-separated episodic representations.

However, a null result for neurons labeled as “Type I” does not constitute strong evidence against a simpler explanation according to which the latencies for all 21 pair-coding neurons were longer for the Np stimuli than the p stimuli. Unless statistical power was very high, the so-called Type I neurons might simply reflect a failure to detect a true latency difference for these neurons as well. The idea that there are two distinct types of neurons based on the latency measures implies that the 21 p-Np latency difference-scores constitute a mixture distribution, one with a mean difference centered on 0 (Type I neurons) and another with a true mean difference greater than 0 (Type II neurons). However, no test for a mixture distribution was reported, so we performed such a test on the latency scores for the 21 pair-coding neurons estimated from Figure S4 of Ison et al. (2015). We first fit a single Gaussian distribution (free parameters = one mean and one standard deviation) to the 21 latency difference scores (p latency minus Np latency), and then fit a Gaussian mixture distribution (free parameters = two means, two standard deviations, and a mixing proportion) to the same data. The fits were performed using maximum likelihood estimation, and the quality of the fits was compared using both AIC and BIC to adjust for the difference in the number of free parameters. According to both goodness-of-fit measures, a single Gaussian distribution model provided a better fit than the two-Gaussian mixture model (AIC = 261.35 and BIC = 263.44 for the single-Gaussian model; AIC = 263.46 and BIC = 268.68 for the two-Gaussian mixture model). Thus, there is no evidence for two types of neurons based on the latency data.

In light of the above results, we next simply compared the average latency of the 21 pair-coding neurons in response to the p stimulus vs. the experimentally associated Np stimulus. This approach does not assume a categorical distinction between Type I and Type II neurons. Across all 21 pair-coding neurons, the average latency to the associated Np stimulus (322 ms) was significantly longer than the average latency to the p stimulus (259 ms), p = 0*.*019. This is the expected result if the experimentally learned Np stimulus triggers a thought of the p stimulus, which in turn, activates the concept neuron. According to these results, the activity of the concept neuron in response to the recently associated Np stimulus still reflects semantic memory, not episodic memory. That being the case, the data do not weigh against the notion that episodic memories are coded in non-overlapping neural assemblies (assemblies that were not recorded in this experiment, which only recorded from semantic-memory concept neurons).

**Supplementary Figure 7. Recognition memory procedure used by Faraut et al. (2018)**

*Note*. Recognition memory task experimental design (reproduced and adapted from Faraut et al., 2018 under Creative Commons License 4.0). The published dataset consists of single-unit recordings from the hippocampus and amygdala from 59 epilepsy patients while completing a recognition memory test, for a total of 87 experimental sessions (Faraut et al., 2018, Chandravadia et al., 2020). Each session consisted of an encoding and retrieval phase. During the encoding phase (a), participants studied images (n=100) from five different visual categories: animals, people, cars/vehicles, outdoor scenes/houses, and flowers/food items. Each trial consisted of a delay (1s blank screen), the presentation of an image (1- or 2- seconds), a second delay (0.5s blank screen), followed by a yes/no question to promote encoding (i.e., “Is this an animal?), with unlimited time to respond. (b) Approximately 15 minutes after the encoding phase, participants completed an old/new recognition memory test during the retrieval phase. Each trial consisted of a delay (1s blank screen), the presentation of an image (1- or 2- seconds), a second delay (0.5s blank screen), followed by an old/new recognition judgement with confidence ratings (1-6) with unlimited time to respond) A total of 100 images were shown; 50 were previously studied during the encoding phase (“old”; targets) and 50 were not previously studied (“new”; foils).

**Supplementary Figure 8. Schematic of hypothetical single-unit data and example of comparing distribution shapes on quantile-quantile (Q-Q) plots**

**
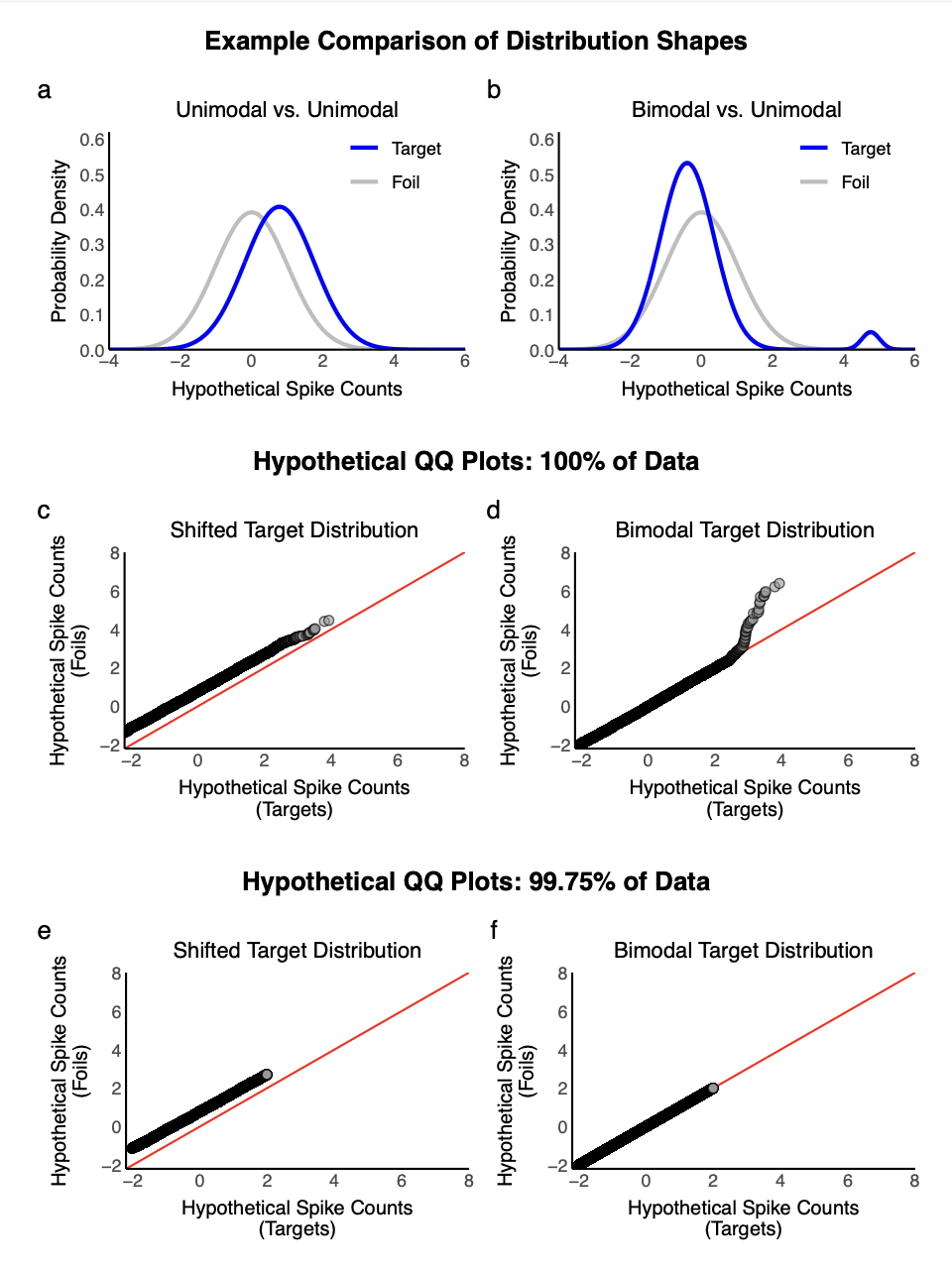
**

*Note.* Schematic illustration of how to interpret empirical quantile-quantile (Q-Q) plots to identify evidence of sparse coding, using a simplified concrete example of single-unit recordings (see Methods under subheading “Statistical Method to Detect the Sparse Coding Signal” for details). The hypothetical dataset consists of 10,000 recordings from 100 single neurons during an old/new recognition task with 100 items (50 old, 50 new). This yields two distributions: 5,000 target-by-neuron responses to old items (100 neurons X 50 old items) and the 5,000 foil-by-neuron responses to new items (100 neurons X 50 new items).

panel (a) shows the probability density functions of the spike-counts from the two hypothetical unimodal distributions: the target-by-neuron response distribution (mean = 0.78 and SD = 0.98; 5,000 recordings) and the foil-by-neuron response distribution (mean = 0 and SD = 1.02; 5,000 recordings). The two distributions share a unimodal shape, but differ in mean and SD. The target distribution is shifted towards the right, indicating an overall higher mean firing rate for target items compared to foil items, a pattern consistent with the generic memory signal which is not item-specific.

panel (b) compares the same foil-by-neuron response distribution from panel (a; mean=0, SD=1.02; 5,000 recordings) to a hypothetical bimodal target distribution containing the sparse coding signal. The lower mode, which contains the majority of target-by-neuron responses, is similar in shape (mean = -0.4, SD = 1; 4,975 recordings) to the foil-by-neuron response distribution. A sparse coding account predicts there would be a small proportion of high firing recordings within the target-by-neuron response distribution, because a singular neuron would fire strongly to only one target item, but not to any of the other target items, nor to any of the foil items. In this simplified example, the sparse coding signal is reflected in the upper mode (mean = 5, SD = 1; 25 recordings, scaled to visualize) of the distribution in panel (b). The presence of the upper mode, reflecting a relatively small number of strong responses from single-neuron responding to few targets, in combination with the lower mode, manifests as an increased positive skew in the target-by-neuron response distribution compared to the foil-by-item distribution. The greater positive skew in the target distribution (relative to the foil) is evidence for sparse coding.

Panels (c) and (d) display empirical Q-Q plots comparing the foil and target distributions from panels (a and b). Spike counts for each neuron (across the entire experiment) in response to each foil and target are rank-ordered separately and plotted on separate axes. Each x-value for a given point on the plot (spike count of a neuron in response to a particular foil) and the corresponding y-value (spike count of a neuron in response to a particular test target) does not necessarily represent the spike counts from the same neuron. In panel (c) the linear pattern shifted from the red line of equivalence reflects the distributions have similar overall shape, in this case unimodal, but the neurons have overall greater firing for targets compared to foils. In panel (d) the Q-Q plot is linear at lower quantiles but deflects upward at upper quantiles, indicating positive skew in the target distribution due to high-firing responses in a subset of neurons, evidence of sparse coding. The majority of points (representing many recordings) are falling on the red line which reflects the majority of neurons respond similarly to targets and foils, thus sharing a similar mean and/or shape. The key difference is the deflection which is characteristic of the sparse coding signal. The visual nonlinear pattern on the Q-Q plot can be statistically confirmed if the target item distribution has increased positive skewness compared to the foil item distribution.

Panels (e) and (f) test this interpretation by removing 0.25% of the highest-firing values from both distributions. In panel (f), removing the highest quantiles from the bimodal (sparse) distribution eliminates the deflection, confirming that sparse coding is driven by a small number of high-firing target responses from a small subset of neurons. In contrast, panel (e) shows no change in the linear pattern from (c), supporting that the difference in (a) was due to a location shift, not sparse coding.

While real neural response distributions are not perfectly Gaussian, the key principle holds: sparse coding predicts greater positive skew in the target-by-neuron distribution, driven by a minority of strong responses. The patterns in panels (d) and (f) exemplify the qualitative and statistical signatures expected if sparse coding is present.
